# Divergent bornaviruses from Australian carpet pythons with neurological disease date the origin of extant *Bornaviridae* prior to the end-Cretaceous extinction

**DOI:** 10.1371/journal.ppat.1006881

**Published:** 2018-02-20

**Authors:** Timothy H. Hyndman, Catherine M. Shilton, Mark D. Stenglein, James F. X. Wellehan

**Affiliations:** 1 College of Veterinary Medicine, School of Veterinary and Life Sciences, Murdoch University, Perth, Australia; 2 Berrimah Veterinary Laboratories, Department of Primary Industry and Resources, Northern Territory Government, Berrimah, Northern Territory, Australia; 3 Department of Microbiology, Immunology, and Pathology, College of Veterinary Medicine and Biomedical Sciences, Colorado State University, Fort Collins, Colorado, United States of America; 4 Department of Comparative, Diagnostic, and Population Medicine, College of Veterinary Medicine, University of Florida, Gainesville, Florida, United States of America; Division of Clinical Research, UNITED STATES

## Abstract

Tissue samples from Australian carpet pythons (*Morelia spilota*) with neurological disease were screened for viruses using next-generation sequencing. Coding complete genomes of two bornaviruses were identified with the gene order 3’-N-X-P-G-M-L, representing a transposition of the G and M genes compared to other bornaviruses and most mononegaviruses. Use of these viruses to search available vertebrate genomes enabled recognition of further endogenous bornavirus-like elements (EBLs) in diverse placental mammals, including humans. Codivergence patterns and shared integration sites revealed an ancestral laurasiatherian EBLG integration (77 million years ago [MYA]) and a previously identified afrotherian EBLG integration (83 MYA). The novel python bornaviruses clustered more closely with these EBLs than with other exogenous bornaviruses, suggesting that these viruses diverged from previously known bornaviruses prior to the end-Cretaceous (K-Pg) extinction, 66 MYA. It is possible that EBLs protected mammals from ancient bornaviral disease, providing a selective advantage in the recovery from the K-Pg extinction. A degenerate PCR primer set was developed to detect a highly conserved region of the bornaviral polymerase gene. It was used to detect 15 more genetically distinct bornaviruses from Australian pythons that represent a group that is likely to contain a number of novel species.

## Author summary

Unlike plants and animals, viruses don’t leave fossilised remains that can be used to study their ancient history. Rarely, however, some virus sequences end up as integrated copies in host genomes. These ‘fossilised’ virus sequences provide a window into virus evolution on a geological time scale. In this study, we identified bornaviruses in pythons, some of which had neurological disease. Bornaviruses have been associated with persistent neurological infections in a variety of mammalian and avian hosts. Here, we reveal that bornaviruses are associated with similar disease in non-avian reptiles as well. These new snake viruses turned out to be most closely related to bornavirus-like sequences that integrated into the DNA of mammals prior to the major extinction event at the end of the age of dinosaurs, the end-Cretaceous (K-Pg) extinction, 66 million years ago, allowing us to infer details of their prehistoric evolutionary history and origin. We also developed a test to detect these viruses from samples collected from live snakes, and used it to identify more related snake bornaviruses, indicating that this group will likely continue to expand.

## Introduction

Bornaviruses are enveloped viruses with single stranded, negative sense, non-segmented, RNA genomes classified to the family *Bornaviridae* in the order *Mononegavirales* [1]. The bornaviral genome is approximately 8,900 nucleotides and is organised into six open reading frames (ORFs): 3‘–N (nucleoprotein)–P (phosphoprotein)–X (accessory protein)–M (matrix protein)–G (glycoprotein)–L (RNA-dependent RNA polymerase)– 5’ [1]. The P and X ORFs overlap; as do M and G [1–4]. Alternative transcription start and stop sites and splicing produce mRNAs that are translated to produce the viral encoded proteins [5–7].

The prototypic bornavirus, Borna disease virus (originally BDV, now BoDV-1), was first detected in horses and sheep [8] but since then, a number of animal hosts have been found to be susceptible to bornavirus infection including rabbits, guinea pigs, macaques, chickens, quail, parrots, canaries, geese, ducks and swans [3, 4, 9–14]. For some time, it was thought that only “warm-blooded” animals (birds and mammals) were susceptible to bornavirus infection [7, 15]. Recent studies have shown that bornaviruses can also infect non-avian reptiles. In 2004, a transcriptome from a Gaboon viper venom gland (*Bitis gabonica*) was reported [16] that contained bornavirus-like sequences [17, 18]. In 2014, a coding complete genome of a bornavirus was recovered from banked frozen tissue from a wild-caught Loveridge’s garter snake (*Elapsoidea loveridgei*), but no clinical or other data was associated with this sequence [19]. The host specificity, association with disease, zoonotic potential, and prevalence of these reptile bornaviruses is totally unknown.

Recommendations for how to taxonomically classify bornaviruses were recently suggested by Kuhn et al. [20] and these have largely been adopted by the International Committee on Taxonomy of Viruses (ICTV) [21]. Concordantly, *Bornaviridae* currently consists of a single genus, *Bornavirus*, which contains eight species: the type species, *Mammalian 1 bornavirus* (formerly *Borna disease virus*); *Mammalian 2 bornavirus*; a snake bornavirus species, *Elapid 1 bornavirus*; and five avian bornavirus species, *Passeriform 1 bornavirus*, *Passeriform 2 bornavirus*, *Psittaciform 1 bornavirus*, *Psittaciform 2 bornavirus*, and *Waterbird 1 bornavirus*.

Endogenous bornavirus-like elements (EBLs) are the result of integration of bornavirus RNA (most likely mRNA) into the host cell’s genomic DNA. This process is probably mediated by long interspersed nuclear elements-1 (LINE-1) retrotransposons [22, 23]. If integration occurs in a germ cell, an EBL can, over time, become fixed in a population. These EBLs then become “fossilised” remnants of ancient infections, and provide rare but informative phylogenetic signals with which to study RNA virus evolution. EBLs have been described in a number of vertebrate and invertebrate genomes [17, 22, 23], including in snake genomes [24]. It is estimated that endogenous bornavirus-like N fragments 1 and 2 (EBLN1-2) integrated into the genomes of six species of viper (from *Crotalus* and *Agkistrodon*) approximately 13 million years ago [24]. In some hosts, the integration of EBLs is estimated to have taken place as long ago as 83.3 million years [25]. The predicted nucleoprotein amino acid sequences of endogenous and exogenous bornaviruses have been compared phylogenetically [17, 24]. EBL-encoded proteins and PIWI-interacting RNAs (piRNA) have been associated with functional roles in antiviral defense and cell cycle progression [25–30]. EBLs are significantly more diverse than known extant bornaviruses, so many EBLs have been presumed to be remnants of extinct bornavirus lineages.

In this study, we characterise the genomes of two bornaviruses from Australian carpet pythons (*Morelia spilota*) with neurological disease. These genomes have features typical of bornaviruses, but have a rearranged gene order that sets them apart from other bornaviruses and mononegaviruses in general. These new bornaviruses are highly divergent, forming a distinct lineage from known extant bornaviruses, and are in fact more closely related to EBLs in a number of mammalian genomes. Using phylogenetic analyses calibrated to fossil records and host-virus cophylogenies, we were able to date the divergence of extant bornavirus lineages prior to the end-Cretaceous extinction, 66 MYA. Overall, these findings expand the known genetic diversity, and host and geographical range of bornaviruses, and shed additional light on the ancient evolutionary history of this group of viruses.

## Results

### Genome and proteome analyses

**Fig 1:**
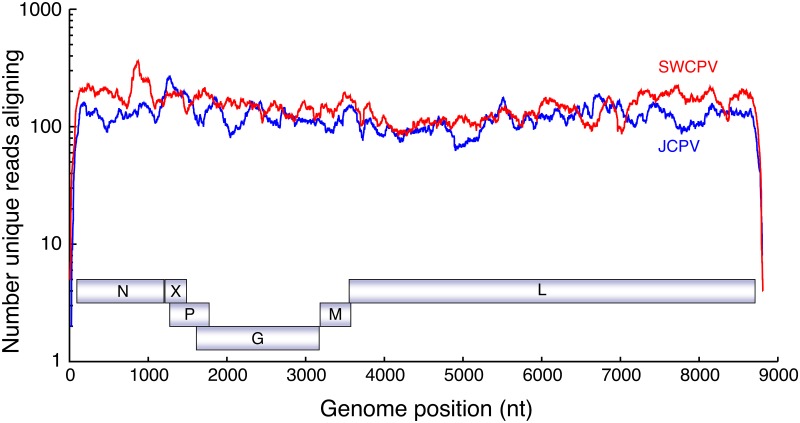
Coverage depth for JCPV and SWCPV genome assemblies. Individual Illumina reads were aligned to the JCPV and SWCPV genome sequences using the Bowtie2 aligner. The number of unique, quality-filtered reads aligning at each base (the coverage) is indicated. Positions of predicted JCPV coding regions are indicated.

**Fig 2:**
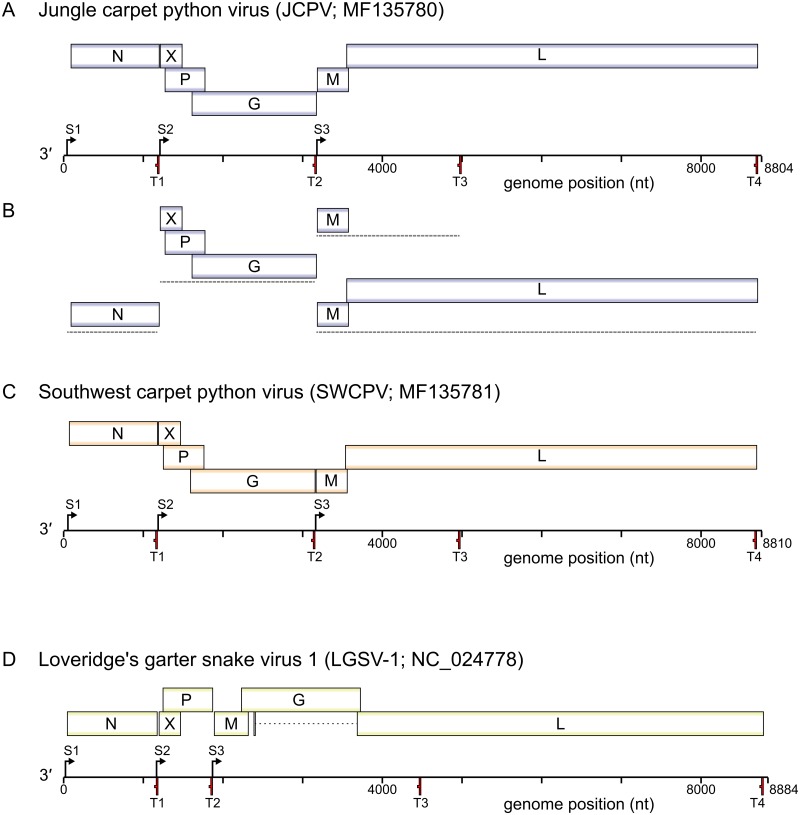
Genome structure and predicted transcriptional map of reptile bornaviruses. (A) to-scale cartoon showing genomic organization of JCPV, including predicted genes and transcriptional start (S) and termination (T) sites. (B) Major predicted transcription products from JCPV genome. (C,D) cartoons of SWCPV and LGSV-1 as in (A).

We used metagenomic sequencing to assemble the coding complete genomes of two distinct bornaviruses from two carpet pythons with neurological disease. The mean coverage for the two genome assemblies was 120 (Carpet python 1, Jungle carpet python virus, JCPV) and 152 (Carpet python 2, Southwest carpet python virus, SWCPV) (Fig 1). The JCPV and SWCPV genomes have six open reading frames (ORFs) larger than 250 nucleotides in the predicted coding orientation (Fig 2). As in other bornavirus genomes, some of the ORFs are partially overlapping. The ORFs encode predicted proteins that correspond to the six major bornavirus proteins but are in the order: 3’–N–X–P–G–M–L– 5’. This gene order differs from that of previously described bornaviruses (N-X-P-M-G-L), with the position of the G and M genes being swapped. The genome assemblies of JCPV and SWCPV contain 93 and 71 nt of predicted noncoding region at their 3’ ends and 50 and 77 nt at their 5’ ends, respectively.

**Fig 3:**
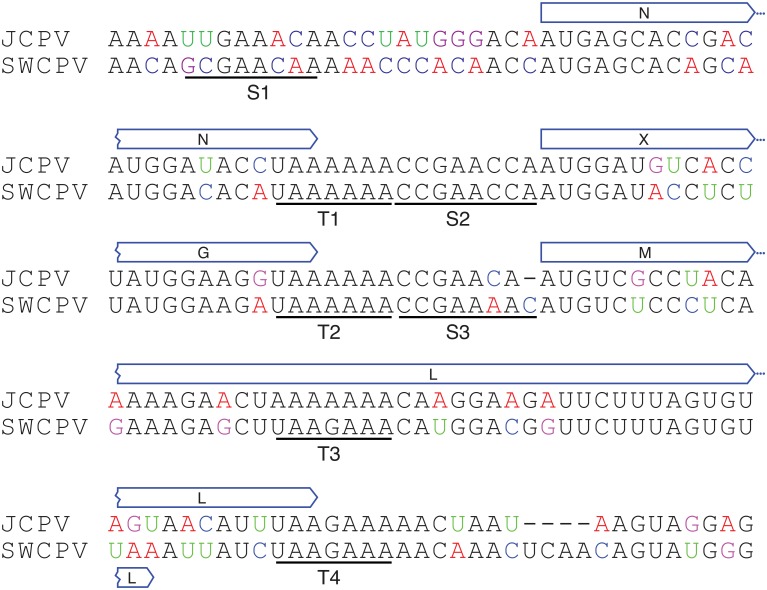
Predicted transcription initiation and termination sites in JCPV and SWCPV genomes. Predicted transcription start (S1-S3) and termination/polyadenylation (T1-T4) signal sites in the JCPV and SWCPV genomes are shown, as are the positions of flanking open reading frames. Sequences are shown in the antigenomic 5′-to-3′ orientation.

We identified putative transcription initiation and termination sites in the JCPV and SWCPV genomes (Fig 2). The consensus sequence of the predicted transcription termination sites in the JCPV and SWCPV genomes is UAA[A/G]AAA (antigenomic 5′-to-3′ orientation) (Fig 3). These termination sites are located at the ends of the predicted N, G, and L coding sequences (CDS) as well as within the L CDS. To identify possible transcription initiation sites, we used the MEME software and an approach similar to that used to identify transcription initiation and termination motifs in nyamiviruses [31, 32]. We identified a shared motif with consensus sequence CCGAA[A/C][A/C]A that may represent transcription initiation sites upstream of the predicted N, X, and M start codons (Fig 3). Transcription from JCPV and SWCPV genome templates is predicted to produce at least 3 or 4 mRNAs (Fig 2B). Although we did not identify evidence of polyadenylation in NGS reads, other bornaviruses and mononegaviruses produce capped, polyadenylated mRNAs, thus we presume that these mRNAs are capped and polyadenylated. We did not find evidence for mRNA splicing in our NGS datasets.

The first ORF in JCPV and SWCPV encodes the predicted viral N proteins, which are 370 (JCPV) and 371 (SWCPV) amino acids (AA), a length within the range of other bornavirus N proteins (369–379 AA). The JCPV and SWCPV N proteins share a 79% pairwise global amino acid identity with each other, 31–45% with endogenous N sequences in mammal genomes, and 17–20% with the N proteins of other bornaviruses (S1 Fig).

The second ORF in JCPV and SWCPV is predicted to encode the viral X protein of 91 (JCPV) and 92 (SWCPV) AA, respectively, which is nearly the same length as the X protein of other bornaviruses, which range from 86–89 AA. JCPV and SWCPV X are 55% identical and possess low but detectable (15–23%) sequence identity with other bornavirus X proteins (S2 Fig).

The third ORF in JCPV and SWCPV may encode the functional equivalent of the phosphoprotein of other bornaviruses. At 167 and 170 AA, respectively, these putative Ps are slightly shorter than the P proteins of other bornaviruses (~200 AA). JCPV and SWCPV P share 74% pairwise amino acid identity and 15–20% with other bornavirus P proteins (S3 Fig).

Open reading frames 4–6 in JCPV and SWCPV encode the predicted G, M, and L of these viruses. These homolog assignments were based on predicted sequence and structural similarity (S4–S6 Figs). The predicted G proteins of JCPV and SWCPV are 76% identical and share 25–29% identity with other bornaviruses, and the top hits from a BLASTP search of the NCBI nr database were to bornavirus G proteins (E values < 1x10^-40^). The predicted M proteins of JCPV and SWCPV are 81% identical and share 22–25% identity with the M of other bornaviruses. The top hit from an hhpred search using JCPV and SWCPV predicted M sequences as queries was to the BDV M protein [E-value 3.5x10^-4^ [33, 34]]. The predicted L proteins of JCPV and SWCPV share 76% pairwise amino acid identity and ~35% with the L proteins of other bornaviruses, making them the most conserved bornavirus proteins.

A number of functionally relevant features have been identified in bornavirus proteins, including nuclear localization and export signals, signal peptides, transmembrane domains, protein-protein interaction domains, and glycosylation sites. We inspected multiple sequence alignments of JCPV, SWCPV, and other bornavirus proteins to look for conserved functional motifs. The degree of sequence similarity between JCPV and SWCPV and other bornavirus protein sequences is low, and in most cases, functionally characterized protein motifs were not obviously conserved (S1–S6 Figs). We therefore also analysed protein sequences using a suite of analysis tools to identify putative functional motifs de novo.

As in other bornaviruses, the G proteins of JCPV and SWCPV contained predicted signal peptides, transmembrane domains, and glycosylation sites [6, 35]. Signal peptides were identified near the C-termini of JCPV and SWCPV G proteins, with predicted cleavage sites after residues 16 and 20, respectively (S4 Fig) [36]. Transmembrane domains were predicted in JCPV and SWCPV G proteins at residues 492–514 and 493–515, respectively. No other JCPV and SWCPV proteins contained predicted signal peptides or transmembrane domains. Multiple N- and O-linked glycosylation sites were predicted in JCPV and SWCPV G [37].

**Fig 4:**
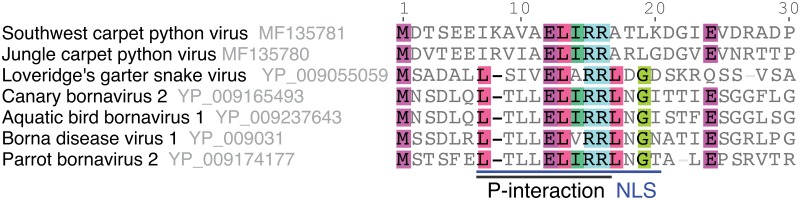
Part of a P-interaction motif is conserved in bornavirus X proteins. A multiple sequence alignment of the putative JCPV and SWCPV X proteins with representative bornavirus X protein sequences. Each virus name is followed by its GenBank accession number. Residues necessary for BoDV-1 X-P interaction and a NLS in BoDV-1 X are highlighted.

Nuclear localization and export signals (NLS and NES) are short motifs that direct proteins into and out of the nucleus [38]. Bornaviruses replicate and transcribe their genomes in the nucleus of infected cells [39], and NLS and NES in bornavirus proteins direct the trafficking of bornaviral ribonucleoprotein complexes [40, 41]. NLS have been identified in the BoDV-1 N, P, and X proteins [41–46]. None of these NLS are obviously conserved in the corresponding JCPV and SWCPV proteins (except for X, see below, Fig 4 and S1–S3 Figs), nor did NLSmapper or NLStradamus predict NLS in these regions. However, these tools both predicted a NLS at another position in JCPV and SWCPV P (58-KKIKRKRETT in JCPV P, and 59-KKAKRKREIV in SWCPV P; S3 Fig).

Nuclear export signals have been identified in bornavirus N and P proteins [40, 47]. These sequences are not well conserved in the JCPV and SWCPV N and P proteins (S1 and S2 Figs). A putative NES was predicted by both NESMapper and WREGX at the same location in JCPV and SWCPV P (in JCPV P: V-89-DSIISKIDSLALL; S3 Fig).

A number of domains that mediate the interaction between bornavirus proteins and cellular proteins (e.g. HMGB1) or between bornavirus proteins have been identified [41, 48–52]. For instance, regions in BoDV-1 N and P that mediate their interaction have been identified [48, 50]. These previously identified motifs are for the most part in regions of weak conservation, making it difficult to extrapolate functional relevance (S1 and S3 Figs).

There were; however, some relatively conserved regions in JCPV and SWCPV proteins with possible functional relevance. In the L protein of JCPV and SWCPV, functional motifs that are widely conserved among mononegavirus RNA-dependent RNA polymerases are present (S6 Fig) [53, 54]. The predicted JCPV and SWCPV X proteins share an EL[I/A/V]RR motif with other bornavirus X proteins (Fig 4 and S2 Fig). This region in BoDV-1 X has been implicated in nuclear localization and interaction with P, and it is possible that this domain mediates similar activities in JCPV and SWCPV X [46, 51, 55].

**Fig 5:**
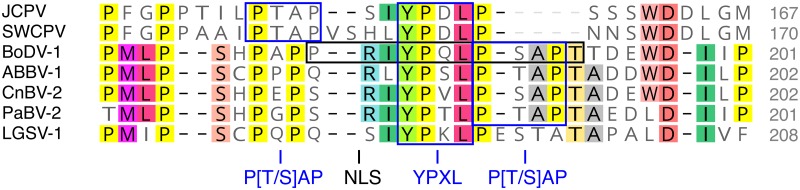
Putative late domain motifs in the C-terminus of bornavirus P proteins. Putative P[S/T]AP and YPXL motifs are indicated in multiple sequence alignments bornavirus P protein sequences. JCPV and SWCPV P were aligned with phylogenetically representative P sequences from the NCBI RefSeq database. Residues conserved in >50% of the sequences are highlighted. Sequence accessions: JCPV: MF135780; SWCPV: MF135781; BoDV-1: NC_001607.1; ABBV-1: NC_029642.1; CnBV-2: NC_027892.1; NC_028106.1; LGSV-1: NC_024778.1. Numbers of C-terminal P residues are indicated.

We also identified a possible functional connection between JCPV and SWCPV P and bornavirus P in the form of putative “late domains” near the C-termini of these proteins (Fig 5). Late domains are short peptide motifs found in a variety of viral proteins that mediate interactions with members of cellular endosomal sorting complexes required for transport (ESCRT) machinery to promote budding of enveloped viruses [56, 57]. The putative late domain sequences in the bornavirus proteins are canonical P[S/T]AP and YPXL motifs, except for the P of Loveridge’s garter snake virus-1 [19], which has a YPXL motif, but not a canonical P[S/T]AP sequence (Fig 5). This region in BoDV-1 P has also been identified as being involved in nuclear import (Fig 5 and S3 Fig) [43, 44].

### Phylogeny

**Fig 6:**
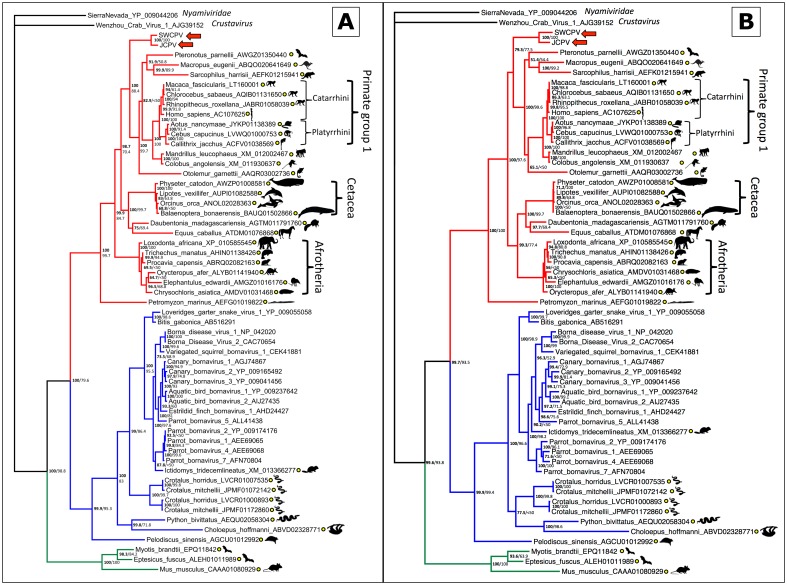
Bayesian phylogenetic tree of MAFFT alignment of homologous bornaviral nucleoprotein (N) amino acid (A) and nucleotide (B) sequences. *Sierra Nevada virus* (GenBank accession # YP_009044206) was used as the outgroup. Confidence of the tree topology is shown by Bayesian posterior probabilities to the left of the slash or above, and maximum likelihood bootstrap values to the right or below. Viruses and EBLs forming a clade with the genus *Bornavirus* are marked in blue, viruses and EBLs clustering with the python bornaviruses are in red, and a third clade of EBLs are in green. Yellow circles indicate EBL sequences.

**Fig 7:**
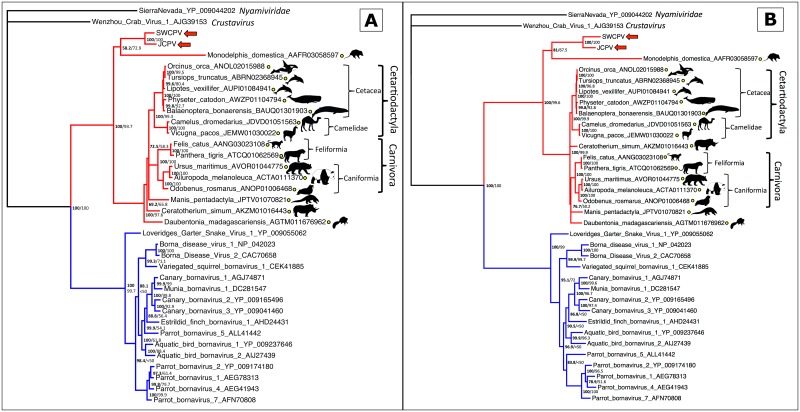
Bayesian phylogenetic tree of MAFFT alignment of homologous bornaviral glycoprotein (G) amino acid (A) and nucleotide (B) sequences. *Sierra Nevada virus* (GenBank accession # YP_009044202) was used as the outgroup. Confidence of the tree topology is shown by Bayesian posterior probabilities to the left of the slash or above, and maximum likelihood bootstrap values to the right or below. Viruses forming a clade with the genus *Bornavirus* are marked in blue, and viruses and EBLs clustering with the python bornaviruses are in red. Yellow circles indicate EBL sequences.

**Fig 8:**
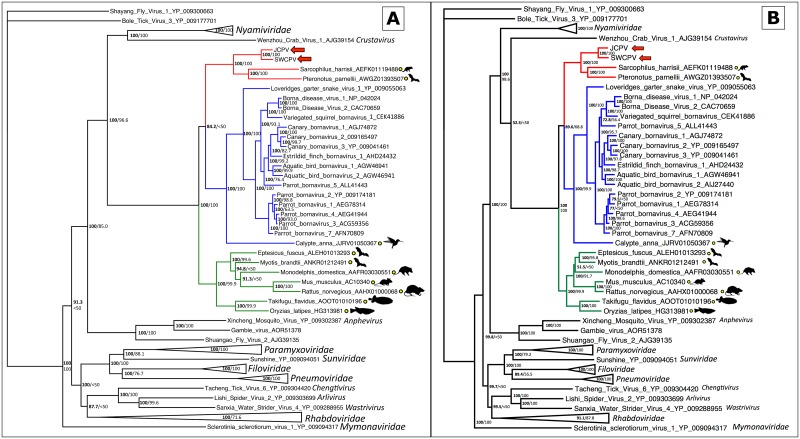
Bayesian phylogenetic tree of MAFFT alignment of homologous bornaviral polymerase (L) amino acid (A) and nucleotide (B) sequences. *Shayang fly virus 1* (GenBank accession # YP_009300663) was used as the outgroup. Confidence of the tree topology is shown by Bayesian posterior probabilities to the left of the slash or above, and maximum likelihood bootstrap values to the right or below. Viruses and EBLs forming a clade with the genus *Bornavirus* are marked in blue, viruses and EBLs clustering with the python bornaviruses are in red, a third clade of EBLs are in green, and non-bornaviral *Mononegavirales* are in black with families collapsed. Yellow circles indicate EBL sequences.

To place these new viruses phylogenetically, we collected sequences of other bornaviruses and of EBLs. Bayesian phylogenetic trees of the bornaviral N, G, and L genes, and their predicted proteins, are displayed in Figs 6, 7 and 8, respectively. Maximum likelihood (ML) phylogenies were also inferred, and their topologies were essentially identical to Bayesian trees. In all phylogenies, JCPV and SWCPV are most closely related to each other, and next most closely related to a clade of EBL sequences found in various mammalian genomes. In the G analysis, this EBL clade contained only a sole marsupial EBL; in the L analysis, it contained a marsupial and a bat EBL; and in the N analysis, this group had EBLs from marsupials, a bat, and a variety of primates, including humans. In the N and G analyses, a clade containing EBLs from laurasiatherian mammals, aye-aye, and in the N analysis, afrotherian mammals, was the next closest. A lamprey EBL was basal to this group in the N analysis.

**Fig 9:**
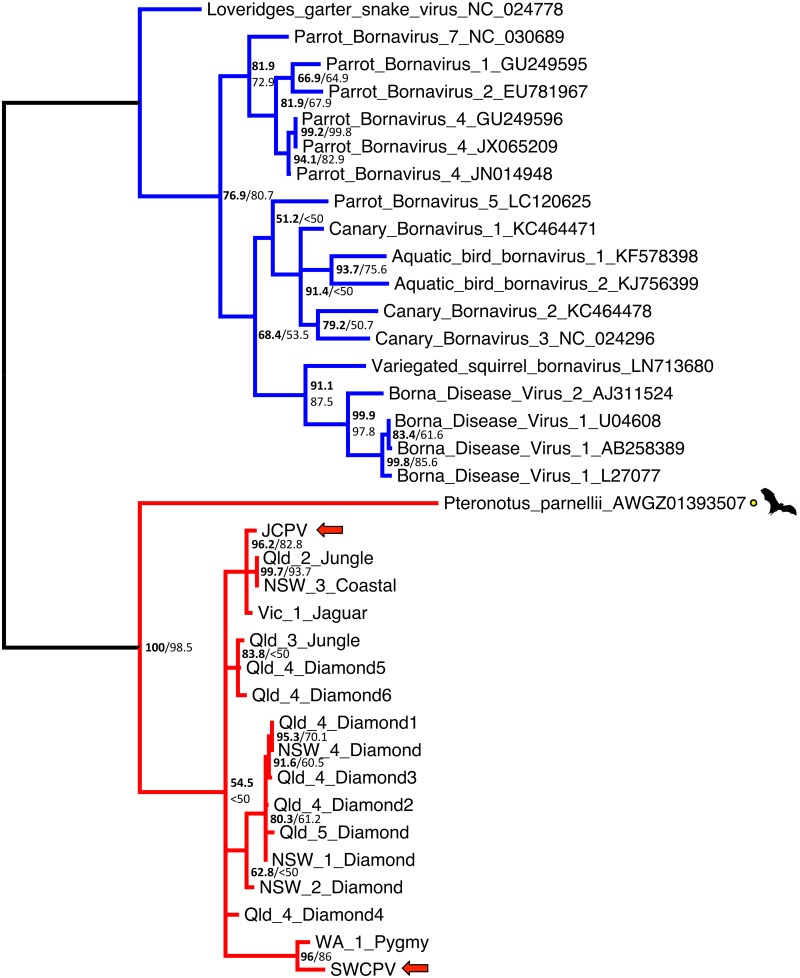
Bayesian phylogenetic tree of MAFFT alignment of homologous bornaviral partial polymerase (L) 211–217 nucleotide sequences. *Loveridge’s garter snake virus 1* (GenBank accession # NC_024778) was used as the outgroup. Confidence of the tree topology obtained is shown by Bayesian posterior probabilities to the left of the slash or above, and maximum likelihood bootstrap values are to the right or below. Viruses in the genus *Bornavirus* are marked in blue, and viruses and EBLs clustering with the python bornaviruses are in red. Python bornaviruses are labelled as Australian state, collection number, host species, and animal number. Qld = Queensland, NSW = New South Wales, Vic = Victoria, WA = Western Australia, Jungle = jungle carpet python (*Morelia spilota cheynei*), Coastal = coastal carpet python (*M*.*s*.*mcdowelli*), Jaguar = jaguar carpet python (*M*.*spilota*), Diamond = diamond python (*M*.*s*.*spilota*), Pygmy = pygmy python (*Antaresia perthensis*).

Using PCR with degenerate primers, 15 distinct bornavirus sequences were detected from 10 other Australian python collections across four Australian states: Queensland, New South Wales, Victoria and Western Australia. From one of these collections (Qld_4), six unique bornavirus sequences were detected from the same species of snake (diamond python, *Morelia spilota spilota*). PCR products were sequenced using Sanger sequencing and phylogenetic analysis of these short L sequences showed diversity was present (Fig 9); however, confidence values for this phylogeny were fairly low.

We next investigated whether the JCPV–and SWCPV–like EBLs were the result of a single integration event into an ancestral mammalian genome or the result of multiple independent integrations. We constructed EBL-host co-phylogenies, and aligned genomic contigs containing EBLs to determine whether EBL integration sites were syntenic. Host-virus/EBL phylogenetic congruence and EBL synteny would be consistent with a single ancestral integration event. We performed these analyses for N–and G–like EBLs.

**Fig 10:**
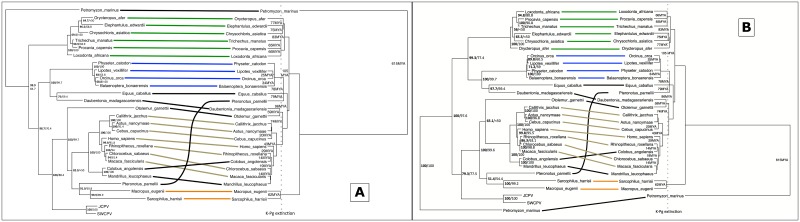
Tanglegram of bornaviral nucleoprotein (N) EBLs (amino acid [A] or nucleotide [B] sequences) and their host species. The nucleoprotein EBL tree is on the left, with confidence of the tree topology obtained shown by Bayesian posterior probabilities to the left of the slash or above, and maximum likelihood bootstrap values to the right or below. The host tree, taken from TimeTree (52), is on the right, and divergence dates in millions of years (MYA) are indicated, with the end-Cretaceous (K-Pg) extinction marked as a dotted line. Connections of an afrotherian clade are marked in green, a cetacean clade are marked in blue, a primate clade are marked in olive, and a marsupial clade are marked in orange.

In host-EBL co-phylogenies, the topologies of the EBL/virus and the host trees were very similar. The predicted N protein analysis found an afrotherian EBL clade with a branching pattern similar to host relationships, a cetacean EBL clade that did not have strong support for branching order, a marsupial EBL clade containing only two members, and a primate clade similar to the branching orders of both platyrhine and catarrhine primates, including humans (Fig 10). Endogenous bornavirus-like elements from two catarrhine primates, colobus (*Colobus angolensis*) and mandrill (*Mandrillus leucophaeus*), clustered separately. An aye-aye (*Daubentonia madagascariensis*) EBL grouped with EBLs from laurasiatherian mammals, and a bat/marsupial EBL cluster grouped with a primate EBL group, both in discordance with the host branching pattern.

Synteny was found for the EBLNs within the afrotherian clade, within the cetacean clade, within “primate group 1”, and between the mandrill and colobus monkey contigs (Fig 10). The aye-aye, Parnell’s mustached bat (*Pteronotus parnelli*), tammar wallaby (*Macropus eugenii*), and Antarctic minke whale (*Balaenoptera bonaerensis*) contigs containing EBLNs were all under 3000bp, and were therefore too short to determine whether synteny was present. Multiple conserved frameshifts and stop codons were present within clades of EBL sequences, consistent with an ancestral integration event and a lack of coding capacity preservation.

**Fig 11:**
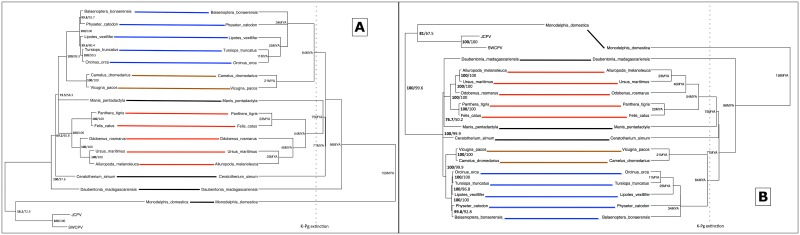
Tanglegram of bornaviral glycoprotein (G) EBLs (amino acid [A] or nucleotide [B] sequences) and their host species. In each panel, The glycoprotein EBL tree is on the left, with confidence of the tree topology obtained shown by Bayesian posterior probabilities to the left of the slash or above, and maximum likelihood bootstrap values to the right or below. The host tree, taken from TimeTree [117], is on the right, and divergence dates in millions of years (MYA) are indicated, with the end-Cretaceous (K-Pg) extinction marked as a dotted line. Connections of a cetacean clade are marked in blue, a camelid clade are marked in brown, and a carnivore clade are marked in red.

The predicted G protein analysis found concordance with the host tree of the EBLs (Fig 11). All Laurasiatherian hosts were in concordance.

**Fig 12:**
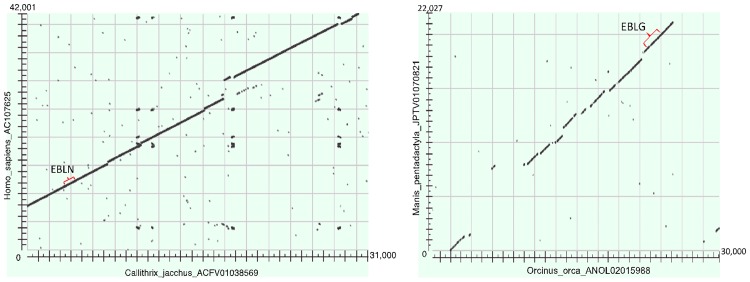
Representative dotplots of genome regions containing endogenous bornavirus-like regions. Human (*Homo sapiens*) vs. marmoset (*Callithripx jacchus*) is shown on the left, with a nucleoprotein EBL marked with a red bracket. Pangolin (*Manis pentadactyla*) vs. orca (*Orcinus orca*) is shown on the right, with a glycoprotein EBL marked with a red bracket. In both cases, the surrounding genome regions also align well, indicating that the EBLs have been introduced into the same site in the two genomes.

Looking at the synteny of EBL-containing genome contigs, all EBLGs from laurasiatherian hosts shared a syntenic integration site, consistent with a single integration event (Fig 12). The aye-aye contig containing an EBLG was only 2273bp, and was therefore too short to determine whether synteny was present. Examination of the G alignments also revealed that frameshifts and stop codons were shared within clades, including the presence of a stop codon homologous to amino acid 113 of JCPV in all laurasiatherian EBLs except tigers (*Panthera tigris*) and polar bears (*Ursus maritimus*) (S1 Appendix). Given that domestic cats (*Felis catus*), giant pandas (*Ailuropoda melanoleuca*), and walruses (*Odobenus rosmarus*), as well as cetartiodactyls and white rhinoceros (*Ceratotherium simum*), all have the stop codon, the most parsimonious explanation is that the stop codon was acquired in an early laurasiatherian and lost in tigers and polar bears independently. Synteny was not found between the gray short-tailed opossum (*Monodelphis domestica*) EBLG contig and other EBLG contigs.

**Fig 13:**
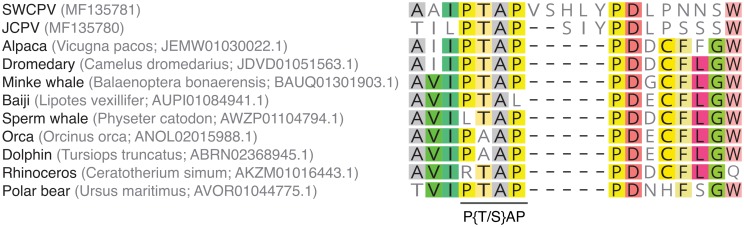
P–like sequences at the beginning of EBLGs. The sequences predicted to be encoded in the other (non G-encoding) reading frames at the beginning of EBLGs were investigated. A motif similar to the end of the JCPV and SWCVP P sequences was identified, consistent with a P–G gene overlap in the virus genomes from which these EBLs derived.

We took advantage of the overlapping ORFs in bornavirus genomes to attempt to infer the gene order in the genomes of the viruses whose integration produced the mammalian EBLs most closely related to JCPV and SWCPV. In JCPV and SWCPV, the beginning of the G ORF overlaps with the end of the P ORF (Fig 2). In contrast, in other bornaviruses and mononegaviruses, the beginning of the G ORF overlaps with the end of the M ORF. We inspected the sequences at the beginning of the G ORFs in the EBLGs and found encoded protein sequences in the other 2 reading frames similar to the ends of JCPV and SWCPV P, including a PTAP late domain motif conserved in most of the sequences (Figs 5 and 13). This supports, but does not prove, that the genome of the ancestor virus of JCPV, SWCPV, and the mammalian EBLs contained the atypical N-X-P-G-M-L gene order.

### Animals

Of the 27 pythons (*Morelia spilota* and *Antaresia* spp.) that were PCR-positive for reptile bornaviruses and in which tissues were examined histologically, 12 had neurological signs that included progressive caudal paresis, flaccid paralysis, delayed righting reflex and incoordination. Of these 12, four had mild to moderate nonsuppurative encephalitis characterised by patchy gliosis with variable lymphocytic perivascular cuffing. These four snakes also had mild to moderate lymphocyte infiltration of a few to many peripheral and autonomic nerves and ganglia. Of 15 PCR-positive snakes without neurological signs, two had mild nonsuppurative encephalitis.

## Discussion

Here, we describe the identification of novel bornaviruses, jungle carpet python virus (JCPV) and southwest carpet python virus (SWCPV), in Australian carpet pythons with neurological disease. These viruses are more closely related to a clade of ‘fossilised’ endogenous bornavirus sequences than they are to other bornaviruses. Thus, JCPV and SWCPV are the ‘living’ descendants of viruses whose integration millions of years ago into ancestral mammalian genomes gave rise to these EBLs. Three major clades of bornaviruses and EBLs have been described: the first described clade, containing members of the genus *Bornavirus* and EBLs; the JCPV/SWCPV clade; and a third clade of N–and L–like EBLs in mammal and fish genomes (Figs 6 and 8). It is plausible that there are extant bornaviruses in this third clade awaiting discovery.

A taxonomic proposal has been submitted to the International Committee on Taxonomy of Viruses supporting the classification of these viruses into a new genus, within the family *Bornaviridae* (TaxoProp 2017.005M.N.v1.Carbovirus). This will provide impetus to resolve the previously identified issue of whether “bornavirus” refers to a member of the family *Bornaviridae* or the genus *Bornavirus*, which contains all previously identified bornaviruses [20]. Our study also provides some guidance as to how the endogenous bornaviruses could be classified, should the ICTV decide to include them in future taxonomy.

We developed a degenerate PCR to detect JCPV and SWCPV and used it to recover an additional 15 unique sequences of related virus from Australian pythons, indicating that this group is likely to contain a diverse group of novel bornaviruses. We were able to detect viral RNA from oral-cloacal swabs, whole blood and a variety of tissue samples (e.g. brain, lung, liver, kidney, gonad). These primers target a conserved region of the L gene, making this assay suitable for diagnostic testing and bornavirus discovery in a variety of host species (not just snakes) where bornavirus infection is suspected.

The genome organisation of JCPV and SWCPV is in large part similar to the members of the genus *Bornavirus* [1, 6, 58, 59], although the gene order in JCPV and SWCPV is N-X-P-G-M-L, whereas the gene order in the “classic” bornaviruses and in most mononegaviruses is N-X-P-M-G-L [G or other transmembrane glycoprotein(s)]. It is likely that along the lineage leading to JCPV, SWCPV, and the related EBLs a rearrangement occurred that resulted in the transposition of the G and M genes. That this rearrangement was possible is remarkable, given that in both configurations the G gene overlaps significantly with neighbouring ORFs. Gene order is correlated with transcription level and mutation rate in the mononegaviruses, with 5’ genes transcribed at lower levels and showing lower mutation rates [60]. The G protein is typically the major antigenic determinant in the *Mononegavirales*; the impact of the altered position on relative gene evolution rates merits future investigation.

Bornaviruses use a variety of mechanisms to produce different mRNA and protein species [1, 6, 58, 61–63]. Initiation and termination of transcription at sites spread through the genome produce monocistronic and polycistronic polyadenylated messages. Additional mRNA species are generated through transcriptional read-through of termination signals and splicing, which enable the translation of downstream ORFs and the production of alternative protein isoforms. Jungle carpet python virus and SWCPV appear to employ a strategy that is generally the same as that used for other bornaviruses to direct the synthesis of viral gene products, as we were able to identify putative transcription initiation and termination sites in their genomes at the expected locations. We did not find evidence for mRNA splicing and could not identify putative splice sites by comparison to other bornaviruses given the high degree of divergence. Nevertheless, it is possible that some of the JCPV and SWCPV mRNAs are spliced. These predictions will have to be validated experimentally once these viruses have been isolated or rescued using reverse genetics.

Dating of ancient viral evolution is problematic. Unlike plants and animals, a fossil record is absent. One approach to estimating viral divergence times has been to calculate rates of change based on isolates from different time points, and to use these rates to predict divergence times of isolates. Using these methods in the order *Mononegavirales*, morbillivirus substitution rates have been estimated to be approximately 6–7 x 10^−4^ substitutions/site/year, predicting divergence dates less than 2000 years ago in the genus [64]. Rhabdoviral substitution rates have been estimated to be approximately 1–8 x 10^−4^ substitutions/site/year [65]. Bornaviral N gene divergence rates have been calculated to range from approximately 0.4–2 x 10^−4^ substitutions/site/year in BoDV-1 to 5–30 x 10^−4^ substitutions/site/year in avian bornaviruses, with the BoDV-1 most recent common ancestor (MRCA) estimated at 302 years ago and the avian bornavirus MRCA at 772 years ago [66]. However, substitution rates may vary significantly. There is evidence of a 27-fold decrease in substitution rates in persistently infected Ebola virus cases [67].

When virus endogenisation appears to predate host divergence, host divergence times can be used to estimate viral divergence, often resulting in much lower rates than those calculated from virus isolates. Endogenous bornavirus integrations in rattlesnakes have been estimated to be 8–13 million years old [24]. Bats in the genus *Eptesicus* have harboured an endogenous bornavirus-like element for 11.8 million years [54]. Bracoviruses, large double-stranded DNA viruses, have been reported to have coevolved with their insect hosts for 310 million years [68], and retroviruses appear to have codiverged with their vertebrate hosts at least 450 million years ago [69]. The discrepancy between higher short term viral evolutionary rates and lower long-term rates appears to be consistent across diverse viral groups [70]. Short term rates may not account for purifying selection seen over longer time scales; this time dependent rate phenomenon needs to be considered when examining viruses over diverse time scales.

The finding that these novel divergent exogenous bornaviruses cluster with an ancestral laurasiatherian EBLG integration (77MYA) and a previously identified ancestral afrotherian EBLN integration (83 MYA, [25]) argues that these viruses diverged from previously known bornaviruses prior to the end-Cretaceous (K-Pg) extinction 66 million years ago, the major extinction event marking the end of the large dinosaurs.

Physiology and the fossil record make it surprising that mammals dominated after the end-Cretaceous extinction. Therapsida, the lineage of which mammals are the only remaining members, were the dominant clade in the Permian [71]. The largest extinction in the fossil record, the end-Permian extinction 251 million years ago, was a climate warming event due to atmospheric carbon release [72]. Mammalian renal and respiratory physiology results in greater water loss than that of Sauropsida (reptiles, including birds) [73, 74]. In the hot arid environment of the end-Permian extinction, the mammalian ancestors were severely reduced in number and size, and reptiles dominated [75].

There is strong evidence that climate warming played a significant role in the end-Cretaceous extinction [76]. Given the physiological disadvantages mammals have with water conservation, and the previous result of the end-Permian climate warming, it is unexpected that mammals would dominate following an end-Cretaceous climate warming event.

Endogenous viral elements have been shown to protect against infection with similar viruses, including an EBLN in a thirteen-lined ground squirrel (*Ictidomys tridecemlineatus*) that inhibits replication of Borna disease virus [27, 77]. It is possible that the JCPV–and SWCPV–like EBLs block the replication of other members of this clade. EBLs found in the clade containing JCPV and SWCPV, with the exception of the lamprey EBLN, are all from mammalian hosts.

Diverse members of the *Mononegavirales* have been strongly associated with significant population-level impacts on host species: for example, Ebola virus in central African great apes [78], distemper virus in Ethiopian wolves (*Canis simensis*) [79], morbillivirus infection in pilot whales (*Globicephela melas*) [80], and viral haemorrhagic septicaemia virus in Pacific herring (*Clupea pallasi*) [81].

In this study, it was revealed that infection with JCPV or SWCPV is variably associated with neurological signs, and only occasionally with nonsuppurative encephalitis and peripheral ganglioneuritis. The presence of virus in clinically and neurohistologically normal snakes is similar to what has been described in other species that are susceptible to bornavirus infection [7, 82–84]. In birds infected with avian bornaviruses, in some populations and species, there is a relatively good correlation between presence of virus with neuropathology [85, 86], although in others, the correlation is highly variable, with persistent infection in clinically normal birds being common [12, 14, 87–89]. When bornavirus is associated with neuropathology, nonsuppurative encephalitis, as was seen in our cases, is typical [82, 90, 91]. Also, the nonsuppurative encephalitis seen in the present study was usually accompanied by nonsuppurative peripheral ganglioneuritis; as has been described in birds [83, 85, 86]. Therefore, while these preliminary results suggest that JCPV and SWCPV have an association with disease in pythons, further work is needed to describe a number of fundamental aspects of these viral infections, including epidemiological and controlled infection studies to investigate disease causality and the mechanisms of viral transmission.

The zoonotic potential of bornaviruses remains unclear. Avian bornaviruses (ABV) are not known to naturally infect mammals [92], and ABV genotypes 2 and 4 could not be grown in three mammalian cell lines [93]. There has long been controversy regarding the association of BoDV-1 with human neuropsychiatric disorders [82, 84, 94, 95]. However, in light of the recent association between fatal neurological disease in people and variegated squirrel bornavirus 1 infection that plausibly was acquired from squirrels [96], the zoonotic potential of these novel bornaviruses from pythons should also be examined, though no data exist to suggest that these viruses can infect mammals.

Disease due to bornavirus infection appears to most significantly impact the surviving dinosaurs, the birds. Psittacine bornaviruses have a significant negative impact on populations of the critically endangered Spix’s macaw (*Cyanopsitta spixii*) [97]. The extant Dinosauria appear to have relatively fewer endogenous viruses than mammals, and endogenous bornaviruses appear to be 6 to 13-fold less common in bird genomes than mammals [98]. Endogenous bornaviruses do not show deep relationships within the Dinosauria; one survey of 48 avian genomes found that only three had endogenous bornavirus-like elements, and all three appeared to have independent integration sites and not share ancestry [98]. The prehistoric clinical significance of the JCPV/SWCPV clade of viruses is not known, but protection from bornaviral disease by EBLs could have provided a selective advantage to mammals following the end-Cretaceous extinction.

## Materials and methods

### Ethics statement

Tissue samples from snakes were used in this study. These snakes were humanely euthanased by the attending veterinarians (listed in the acknowledgements section) on humane grounds and due to their poor prognoses. Tissue samples were submitted to the corresponding author and it was only after a number of investigations, were the novel viruses discovered that are described in this manuscript. The decision was then made to disseminate this information by way of publication. For the snakes that were sampled live, they were sampled for the primary purpose of diagnostic screening. This means that the snakes were sampled independent of this study. However, the information obtained from this diagnostic testing was then used for this manuscript. In short, no animal was used for the primary purpose of this study. Animals were sampled and tested for diagnostic purposes so that the attending veterinarian could better manage the snake collections they were working with.

### Animals

In 2013, an adult jungle carpet python (*Morelia spilota cheynei* = Carpet python 1) from a zoological park in Queensland, Australia, was euthanased for ongoing and progressive signs of neurological disease that did not respond to supportive care. In 2014, a male 3.5 year old south-west carpet python (*Morelia spilota imbricata* = Carpet python 2) from a private collection in Western Australia, was euthanased for ongoing inappetance, lethargy and a decreased righting reflex. Fresh frozen brain, lung and spleen from Carpet python 1, and fresh frozen brain from Carpet python 2, were submitted for molecular testing of viruses at Murdoch University, Australia. Once PCR testing was developed to screen for reptile bornaviruses (see elsewhere), other infected pythons were identified through the diagnostic laboratory of one of the authors (THH). All snake samples used in this study were primarily analysed for diagnostic purposes, as per the request of the veterinarians attending to the health of these animals, and who submitted the samples for diagnostic testing. All the veterinarians who submitted samples that were subsequently used in this study were aware that their samples could be used for research purposes.

### Nucleic acid extraction

The three tissue samples from Carpet python 1 were pooled together. Nucleic acid was extracted from tissues using the MELT Total Nucleic Acid Isolation System (cat. no. AM1983, Ambion, Thermo Fisher, Victoria, Australia) following the manufacturer’s recommendations. During the extraction from the pooled tissue homogenate from Carpet python 1, the optional DNase treatment step was included. Extracted nucleic acid from Carpet python 2 was treated with DNase using the TURBO DNA-*free* Kit (cat. no. AM1907, Ambion, Thermo Fisher, Victoria, Australia) following the manufacturer’s recommendations.

### Next generation sequencing and genome analyses

The two DNase-treated total nucleic acid extracts (one from each carpet python) were then depleted of ribosomal RNA (rRNA) using the RiboMinus Eukaryote System v2 (cat. no. A15026, Ambion, Thermo Fisher, Victoria, Australia) following the manufacturer’s recommendations. The DNase-treated rRNA-depleted total nucleic acid extracts were then submitted to the Australian Genome Research Facility (www.agrf.org.au) for next-generation sequencing using Illumina HiSeq 2500 (Carpet python 1) and Illumina MiSeq (Carpet python 2). Both sequencing reactions used 150 base pair, paired-end (PE) reads.

De novo assembly of unfiltered sequencing reads into contiguous sequences (contigs) was performed using CLC Genomics (version 8.0.1, QIAGEN). Standard settings for the assembly were used except the Word Size and Bubble Size were set at 64 and 100, respectively. The assembled contigs were used as queries to search for similarities in the NCBI database using BLASTN after limiting the search to virus organisms (taxid: 10239). The nucleotide and predicted amino acid sequences of contigs that had similarities to viral sequences were then used as queries to search the NCBI non-redundant nucleotide and protein databases using BLASTN and BLASTP, respectively. These methods identified contigs corresponding to coding complete bornaviral genomes in each of the two carpet python samples. The sequences of these two genomes were checked for accuracy by mapping the reads to the putative bornaviral genomes using standard settings in CLC Genomics.

The accuracy of these genome sequences was then validated using a second approach that also searched for other possible candidate pathogens. Low quality sequences were trimmed or removed using the Trimmomatic tool, version 0.32 [99], with the following settings: LEADING:20, TRAILING:20, SLIDINGWINDOW:4:25, and MINLEN:40. Then, potential PCR duplicated sequences were collapsed using the CD-HIT-EST tool, version 4.6 [100], with parameter–c 0.96. Remaining reads were assembled using the SPAdes assembler, version 3.5.0 [101], with default parameters. To validate bornavirus assemblies, reads were mapped to draft genome assemblies using Bowtie2, version 2.2.5 [102], with parameters—local—qc-filter—score-min C,120,1. Draft assemblies were corrected and validated by manual inspection in Geneious, version 9.0.4 [103]. Depth of coverage was calculated using SAMTOOLS (htslib) version 1.2.1 [104]. Remaining (non bornavirus) contigs and non-assembling reads were then taxonomically assessed, first by using the BLASTN tool to align to the NCBI nucleotide database. Sequences not producing a nucleotide-level alignment were then searched via translated-nucleotide to protein alignments against the NCBI non-redundant (nr) protein sequence database using the Rapsearch2 tool, version 2.23, with parameters–a t, −1 20, and–e1 e-2 [105].

The two genome assembly methods used (CLC Genomics and SPAdes Assembler) produced identical genome sequences. From the tissue homogenate from Carpet python 1, an 8,915 nucleotide contig was assembled that represents a coding complete genome of a bornavirus that we named jungle carpet python virus (JCPV). From the brain homogenate of Carpet python 2, an 8,920 nucleotide contig was assembled that represents a coding complete genome of a second bornavirus that we named southwest carpet python virus (SWCPV). These two genome sequences have been deposited in Genbank under accession numbers MF135780 (JCPV) and MF135781 (SWCPV). Raw sequence data has been deposited in the NCBI Short Read Archive (SRA; accession PRJNA390668).

Homologs of predicted viral protein sequences were identified using BLASTP to search the NCBI (nr) protein database. For sequences that lacked detectable similarity by BLASTP, the HHpred homology detection and structure prediction tool, version 2.0 [106], was also used. To identify endogenous bornavirus-like sequences, the TBLASTN tool was used to search the NCBI genomes database using the predicted bornaviral protein sequences as queries.

We used a variety of tools to perform de novo identification of putative functional features in JCPV and SWCPV genomes and proteins. De novo identification of NLS and NES is prone to false positive predictions [107]. For instance, the NLS in BoDV-1 X resembles a leucine-rich NES more than it does a typical NLS [46]. We therefore used multiple prediction tools and report candidate NES and NLS that were identified at the same positions in both JCPV and SWCPV proteins. We used NLSmapper and NLStradamus to predict NLS, and NESMapper and Wregex to predict NES [107–110]. Transmembrane domains were predicted using the TMHMM2.0 web service [111]. Signal peptides were predicted using the SignalP 4.1 server with the sensitive option enabled [36]. Putative transcription termination sites were predicted based on their genomic location, their conservation in the two new viral genomes, and similarity to well characterised sites in the genomes of other bornaviruses [4, 58, 62]. Transcription start sites were predicted using MEME software (v 4.12.0), using as input to MEME the 20 bases before the predicted N start codons as well as the bases between the poly-A tracts after the N and G ORFS and the predicted start codons of the X and M ORFs [31].

### Development of PCR to detect JCPV and SWCPV in clinical samples

A PCR was designed to detect JCPV and SWCPV. Due to the considerable divergence of these viruses from other known bornaviruses, regions of the RNA-dependent RNA polymerase (L) that are highly conserved among bornaviruses were surveyed for suitable primer annealing targets. The primer pair Reptile bornavirus F (RBV-F) 5’ GGNATGAGRCARAARYTNTGRAC and RBV-R 5’ AARTAYTGYTTYTTNCCRTAYTCRTA was chosen (S7 Fig). These primers were then tested on oral-cloacal swabs, whole blood and a variety of tissue samples obtained from Australian captive pythons, many of which had neurological signs of disease. Nucleic acid was extracted from oral-cloacal swabs using the Purelink Viral RNA/DNA Mini Kit (cat. no. 12280050, Thermo Fisher) according to the manufacturer’s recommendations. For whole blood and tissue samples, the QIAamp *cador* Pathogen Mini Kit (cat. no. 54104, Qiagen) was used. For whole blood, a 2 μL aliquot was added to 198 μL of phosphate-buffered saline (PBS, pH 7.4) and then the manufacturer’s instructions were followed. For tissue samples, nucleic acid was extracted following the manufacturer’s recommendations that included pretreatment method T4. Briefly, segments of tissue with a total volume of ~2–3 mm^3^ were homogenised in phenol (pH 8, cat. no. P4557, Sigma) and PBS using a Mini-Beadbeater 24 (cat. no. 112011EUR, Biospec) at 3,000 oscillations per minute for 2 min using 0.5 mL of zirconia/silica beads (1 mm diameter, cat. no. 11079110z, Biospec). Nucleic acid was eluted into 30 μL of RNase-free water (swabs) or buffer AVE (blood and tissue). For PCR, 10 μL of 2 x master mix and 0.8 μL of RT-Taq enzyme from the Superscript III One-Step RT-PCR System with Platinum *Taq* DNA Polymerase (cat. no. 12574026, Thermo Fisher) were added to RBV-F and RBV-R (1 μM final concentration for each primer). The total volume was brought to 20 μL using 1 μL of extracted nucleic acid and PCR-grade water. Cycling conditions consisted of 50°C x 30min, 94°C x 2min, 15 x (94°C x 20s, 65°C x 30s [decreased by 1°C every cycle], 72°C x 25s), 35 x (94°C x 20s, 50°C x 30s, 72°C x 25s). Ribonucleic acid from JCPV was used as a positive control and a no template extraction was used as a negative control. Amplicons near to the expected size (256 nucleotides) were cut from the gel and sequenced using standard methods. Fifteen unique bornavirus sequences were obtained (GenBank accession numbers MF166903-MF166917) and were included with JCPV and SWCPV in phylogenetic analyses after the primer sequences had been removed.

For 27 Australian pythons that were PCR positive for reptile bornaviruses, a wide range of tissues, including brain, were examined histologically using standard processing techniques and haematoxylin and eosin staining.

### Phylogeny

Predicted JCPV and SWCPV N, G, and L amino acid sequences were used in TBLASTN searches of the GenBank database, including the whole genome shotgun contigs (wgs), to look for endogenous bornaviral elements. Endogenous bornaviral elements were examined for potential frameshifts using BLASTX, and amino acid sequences accounting for this were predicted (S1 Appendix). Homologous predicted viral and endogenous amino acid sequences were aligned using MAFFT [112]. Amino acid alignments were then converted back to the nucleotide sequences using PAL2NAL [113]. Sierra Nevada virus, in the *Nyamiviridae*, was used as an outgroup for the N and G analyses. Due to wider conservation, representatives of all the genera of *Mononegavirales* were included in the L analysis, and Shayang fly virus 1 was designated as the outgroup. Bayesian analyses of nucleotide alignments were performed using MrBayes 3.2.6 on the CIPRES server, with a general time reversible (GTR) model and a proportion of invariant sites [114, 115]. The first 25% of 2,000,000 iterations were discarded as a burn in. Maximum likelihood (ML) bootstrap analyses of each alignment were performed using RAxML on the CIPRES server with gamma distributed rate variation and a proportion of invariant sites and a GTR model. Bootstrap analysis was used to test the strength of the ML tree topology, with 1000 subsets [116].

For analysis of additional shorter python bornavirus partial L sequences, homologous 211–217 nucleotide sequences were aligned based on MAFFT alignments of predicted amino acid sequences. Loveridge’s garter snake virus 1 was used as an outgroup, and Bayesian and maximum likelihood analyses were performed using a GTR model with the above software.

Host trees with divergence times were obtained from TimeTree [117]. Tanglegrams were generated using Dendroscope 3 [118]. To assess synteny of EBL integration sites in host genomes, TBLASTX comparisons of pairs of sequences were analysed using dot plots.

## Supporting information

S1 Fig Alignment of bornavirus N proteins. JCPV and SWCPV N were aligned with phylogenetically representative N sequences from the NCBI RefSeq database. Predicted and validated functional motifs are indicated (see text). Residues conserved in >50% of the sequences are highlighted. Sequence accessions: JCPV: MF135780; SWCPV: MF135781; BoDV-1: NC_001607.1; ABBV-1: NC_029642.1; CnBV-2: NC_027892.1; NC_028106.1; LGSV-1: NC_024778.1.
(TIFF)

S2 Fig Alignment of bornavirus X proteins. JCPV and SWCPV X were aligned with phylogenetically representative X sequences from the NCBI RefSeq database. Predicted and validated functional motifs are indicated (see text). Residues conserved in >50% of the sequences are highlighted. Sequence accessions: JCPV: MF135780; SWCPV: MF135781; BoDV-1: NC_001607.1; ABBV-1: NC_029642.1; CnBV-2: NC_027892.1; NC_028106.1; LGSV-1: NC_024778.1.
(TIFF)

S3 Fig Alignment of bornavirus P proteins. JCPV and SWCPV P were aligned with phylogenetically representative P sequences from the NCBI RefSeq database. Predicted and validated functional motifs are indicated (see text). Residues conserved in >50% of the sequences are highlighted. Sequence accessions: JCPV: MF135780; SWCPV: MF135781; BoDV-1: NC_001607.1; ABBV-1: NC_029642.1; CnBV-2: NC_027892.1; NC_028106.1; LGSV-1: NC_024778.1.
(TIFF)

S4 Fig Alignment of bornavirus G proteins. JCPV and SWCPV G were aligned with phylogenetically representative G sequences from the NCBI RefSeq database. Predicted and validated functional motifs are indicated (see text). Residues conserved in >50% of the sequences are highlighted. Sequence accessions: JCPV: MF135780; SWCPV: MF135781; BoDV-1: NC_001607.1; ABBV-1: NC_029642.1; CnBV-2: NC_027892.1; NC_028106.1; LGSV-1: NC_024778.1.
(TIFF)

S5 Fig Alignment of bornavirus M proteins. JCPV and SWCPV M were aligned with phylogenetically representative M sequences from the NCBI RefSeq database. Predicted and validated functional motifs are indicated (see text). Residues conserved in >50% of the sequences are highlighted. Sequence accessions: JCPV: MF135780; SWCPV: MF135781; BoDV-1: NC_001607.1; ABBV-1: NC_029642.1; CnBV-2: NC_027892.1; NC_028106.1; LGSV-1: NC_024778.1.
(TIFF)

S6 Fig Conserved motifs in block III of JCPV and SWCPV L proteins. An alignment of the region of bornavirus L and EBLL sequences that includes the conserved A, B, C, and D motifs in Block III that were described in Poch et al. [53]. Residues that were found by Poch et al. [53] to be universally conserved are indicated by stars. Consensus residues shared by >50% of the sequences are highlighted. Sequence accessions: JCPV: MF135780; SWCPV: MF135781; BoDV-1: NC_001607.1; ABBV-1: NC_029642.1; CnBV-2: NC_027892.1; NC_028106.1; LGSV-1: NC_024778.1. Alignment corresponds to residues 337–645 of BoDV-1 L.
(TIFF)

S7 Fig Primer annealing sites. A multiple sequence alignment of the putative jungle carpet python virus and southwest carpet python virus L proteins with the cognate L protein sequences from seven of the eight species of the genus *Bornavirus*. This area of the genome has not been sequenced in a *Passeriform 2 bornavirus*. Each virus name is followed by its GenBank accession number and columns are labelled relative to the amino acid residue number of Borna disease virus-1 L protein (AAM68151).
(TIFF)

S1 Appendix Frameshifts of EBLG, EBLL and EBLN with corresponding MAFFT alignments for EBLG and EBLN. EBLG, EBLL and EBLN nucleotide sequences in fasta format followed by amino acid sequences corresponding to frames 1–3.
(ZIP)
