# Supplementary figures and images for: Divergent bornaviruses from Australian carpet pythons with neurological disease date the origin of extant Bornaviridae prior to the end-Cretaceous extinction
Source: PLoS Pathog. 2018 Feb 20;14(2):e1006881. doi: 10.1371/journal.ppat.1006881 (PMC5834213; doi:10.1371/journal.ppat.1006881)

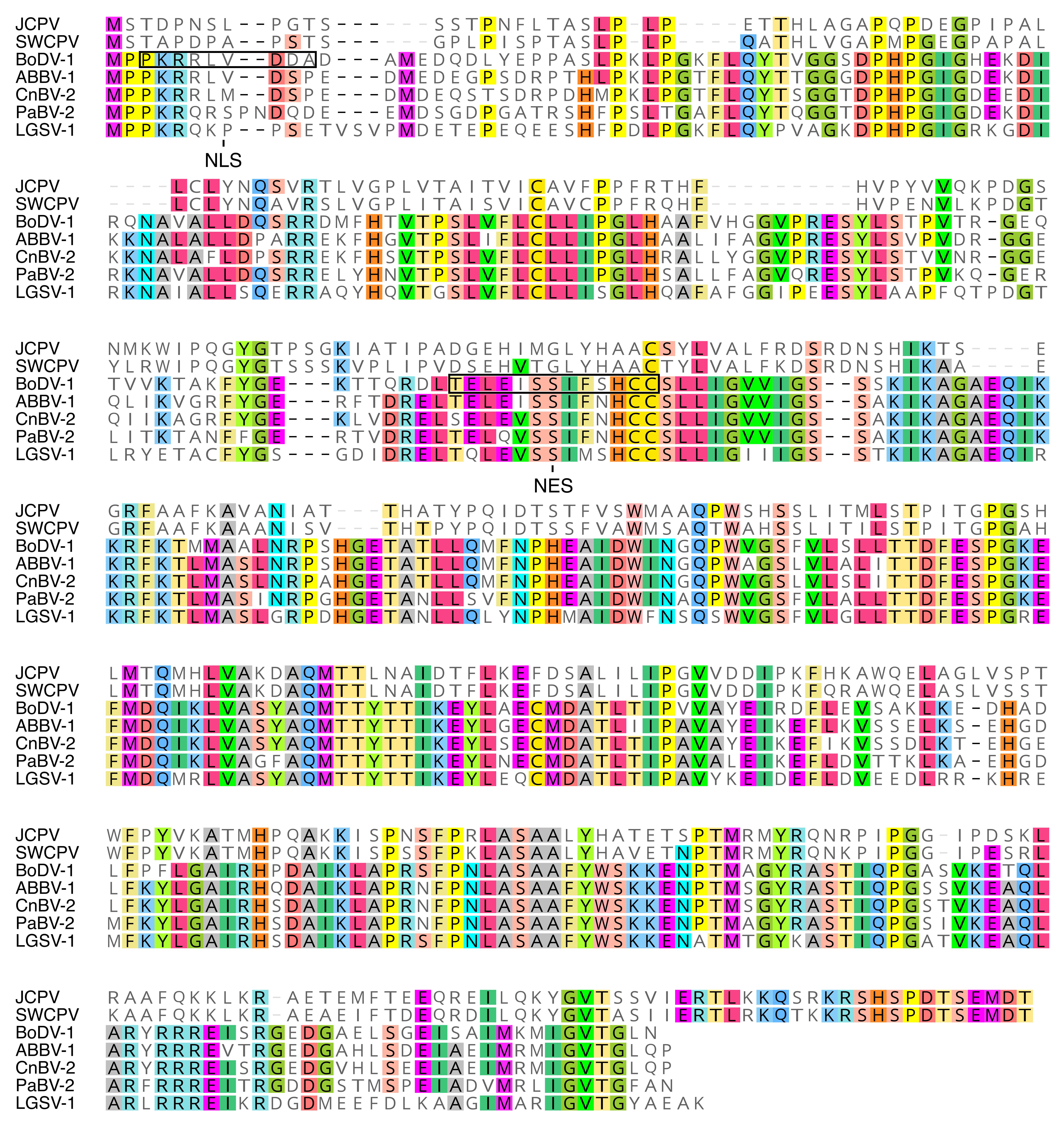

Supplement: S1 Fig — JCPV and SWCPV N were aligned with phylogenetically representative N sequences from the NCBI RefSeq database. Predicted and validated functional motifs are indicated (see text). Residues conserved in >50% of the sequences are highlighted. Sequence accessions: JCPV: MF135780; SWCPV: MF135781; BoDV-1: NC_001607.1; ABBV-1: NC_029642.1; CnBV-2: NC_027892.1; NC_028106.1; LGSV-1: NC_024778.1. (TIFF) [file ppat.1006881.s001.tiff]

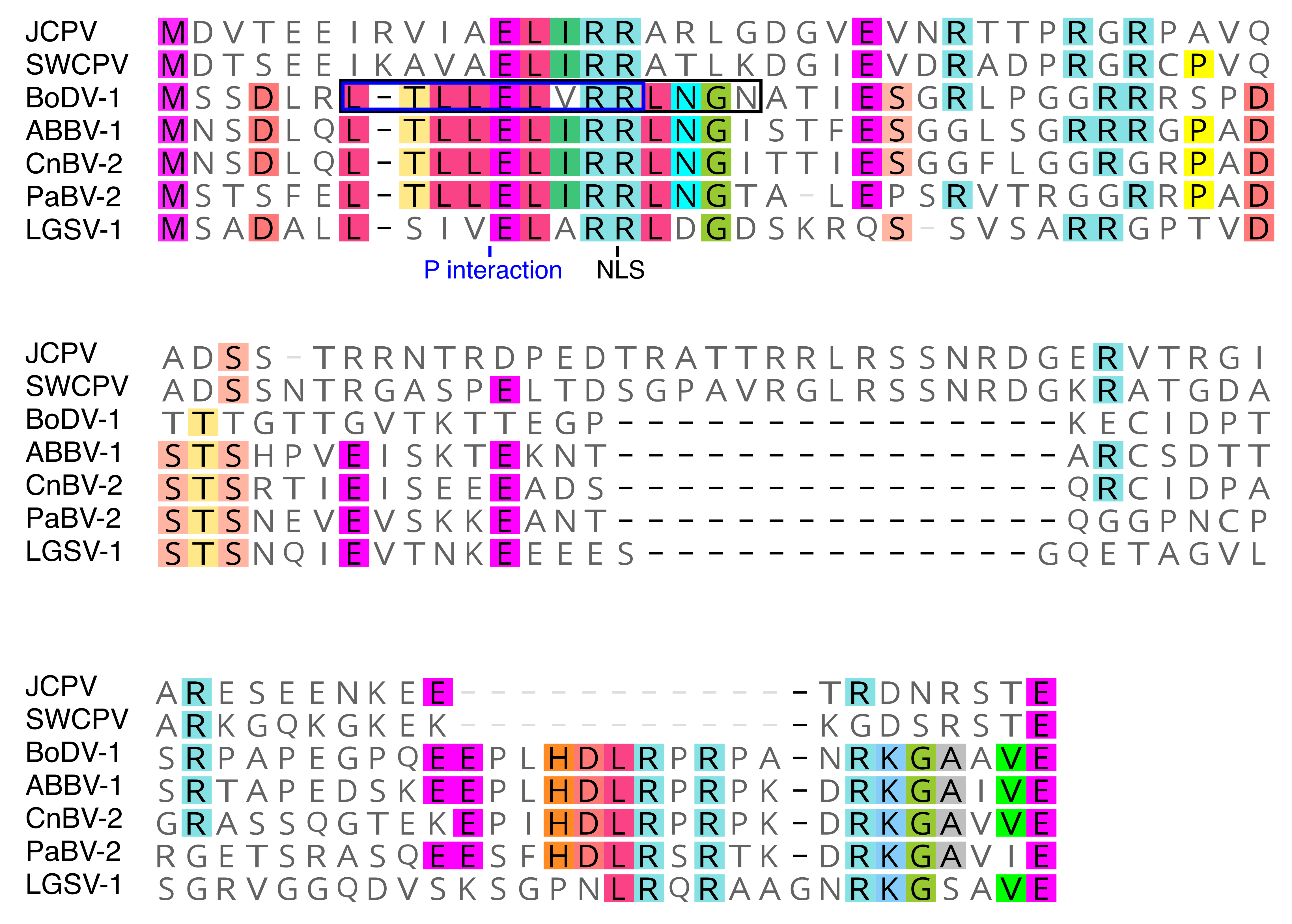

Supplement: S2 Fig — JCPV and SWCPV X were aligned with phylogenetically representative X sequences from the NCBI RefSeq database. Predicted and validated functional motifs are indicated (see text). Residues conserved in >50% of the sequences are highlighted. Sequence accessions: JCPV: MF135780; SWCPV: MF135781; BoDV-1: NC_001607.1; ABBV-1: NC_029642.1; CnBV-2: NC_027892.1; NC_028106.1; LGSV-1: NC_024778.1. (TIFF) [file ppat.1006881.s002.tiff]

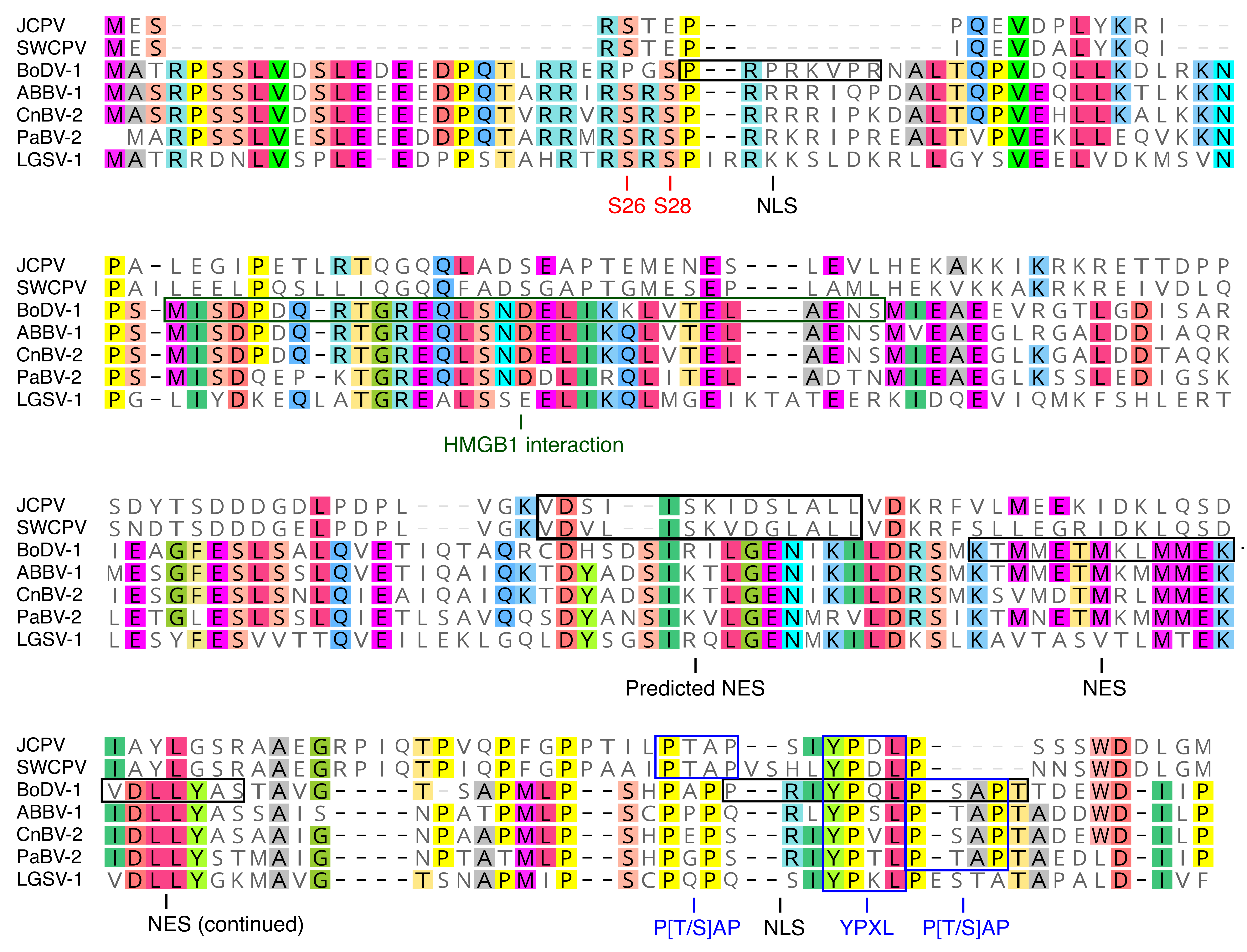

Supplement: S3 Fig — JCPV and SWCPV P were aligned with phylogenetically representative P sequences from the NCBI RefSeq database. Predicted and validated functional motifs are indicated (see text). Residues conserved in >50% of the sequences are highlighted. Sequence accessions: JCPV: MF135780; SWCPV: MF135781; BoDV-1: NC_001607.1; ABBV-1: NC_029642.1; CnBV-2: NC_027892.1; NC_028106.1; LGSV-1: NC_024778.1. (TIFF) [file ppat.1006881.s003.tiff]

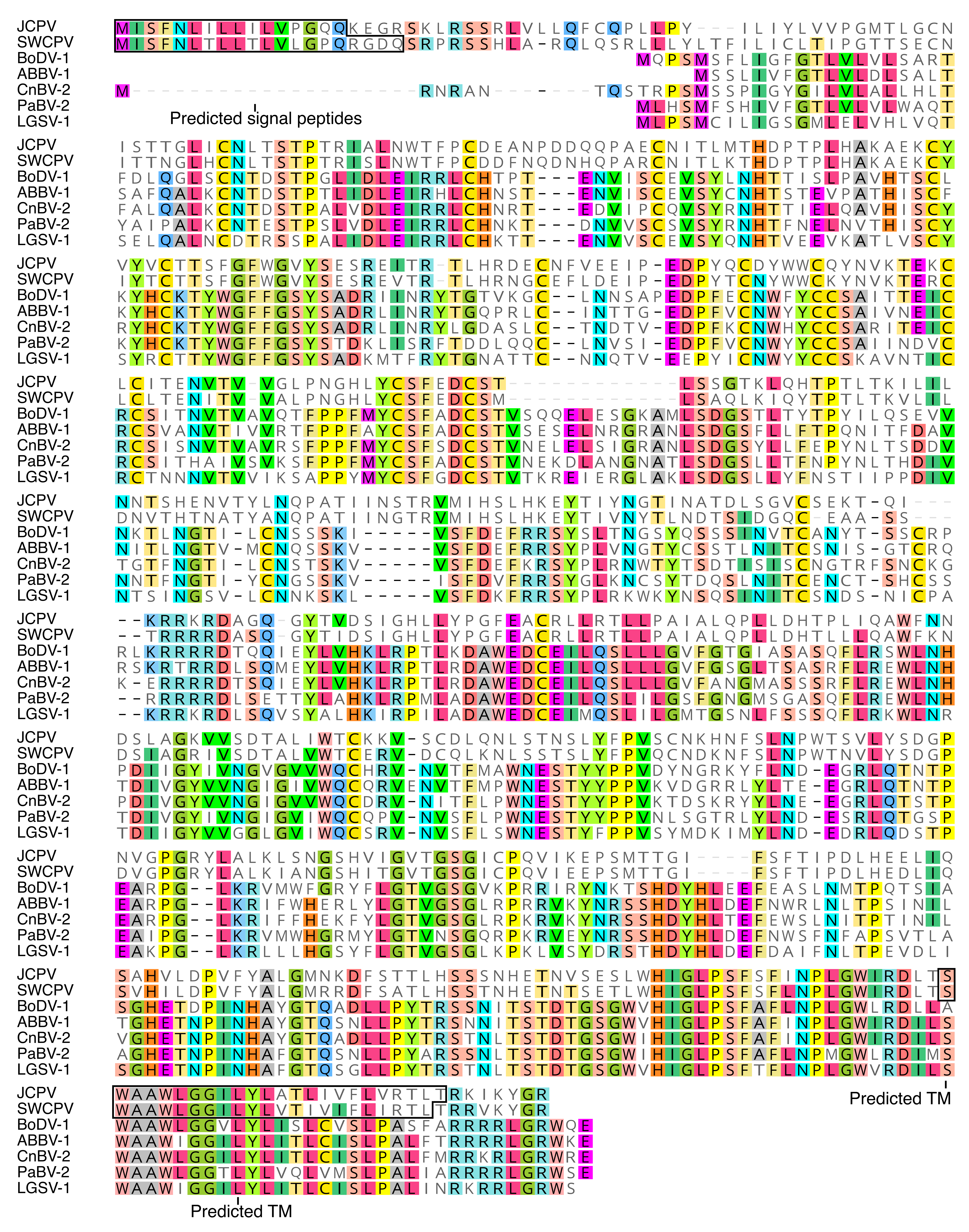

Supplement: S4 Fig — JCPV and SWCPV G were aligned with phylogenetically representative G sequences from the NCBI RefSeq database. Predicted and validated functional motifs are indicated (see text). Residues conserved in >50% of the sequences are highlighted. Sequence accessions: JCPV: MF135780; SWCPV: MF135781; BoDV-1: NC_001607.1; ABBV-1: NC_029642.1; CnBV-2: NC_027892.1; NC_028106.1; LGSV-1: NC_024778.1. (TIFF) [file ppat.1006881.s004.tiff]

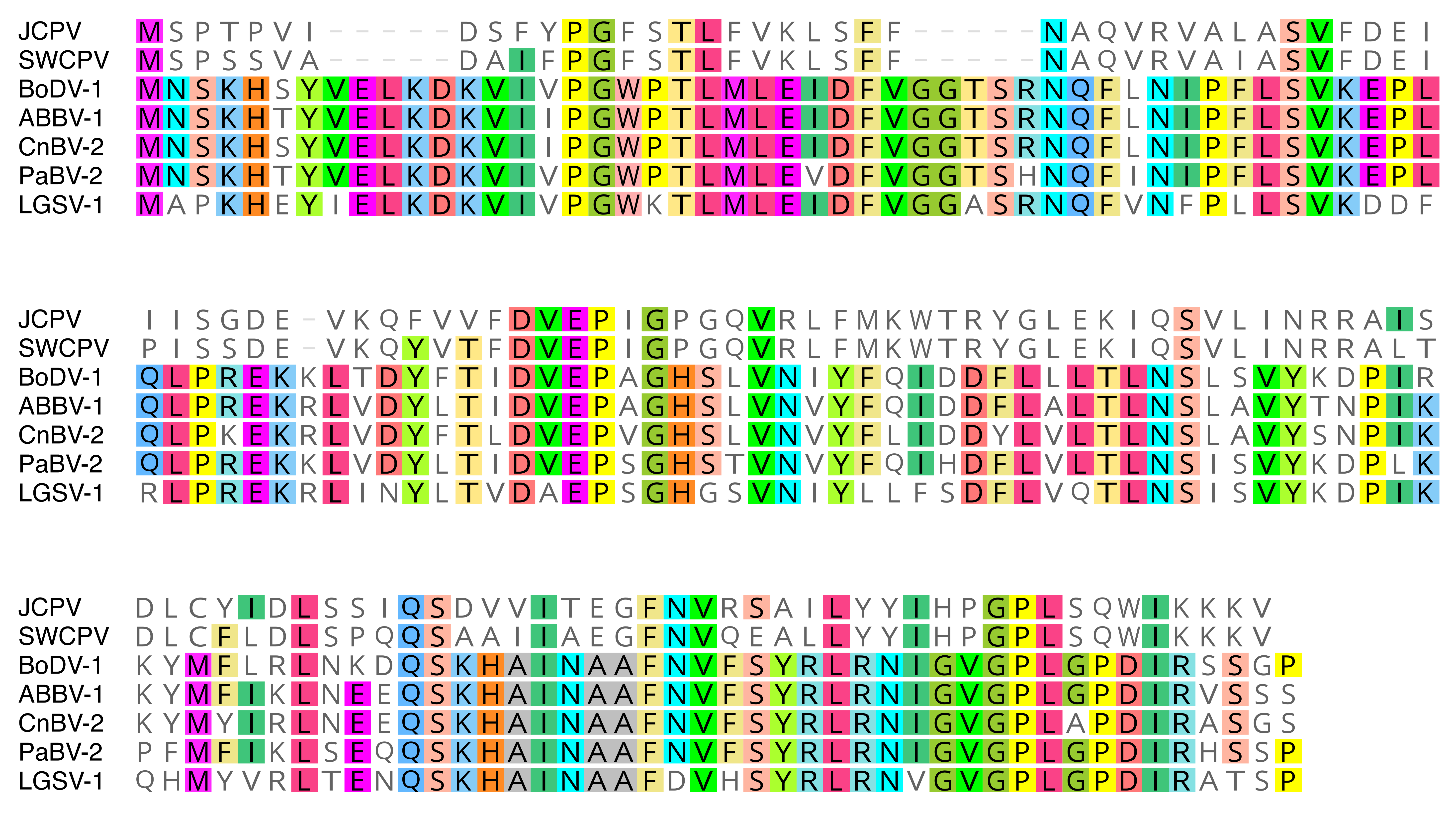

Supplement: S5 Fig — JCPV and SWCPV M were aligned with phylogenetically representative M sequences from the NCBI RefSeq database. Predicted and validated functional motifs are indicated (see text). Residues conserved in >50% of the sequences are highlighted. Sequence accessions: JCPV: MF135780; SWCPV: MF135781; BoDV-1: NC_001607.1; ABBV-1: NC_029642.1; CnBV-2: NC_027892.1; NC_028106.1; LGSV-1: NC_024778.1. (TIFF) [file ppat.1006881.s005.tiff]

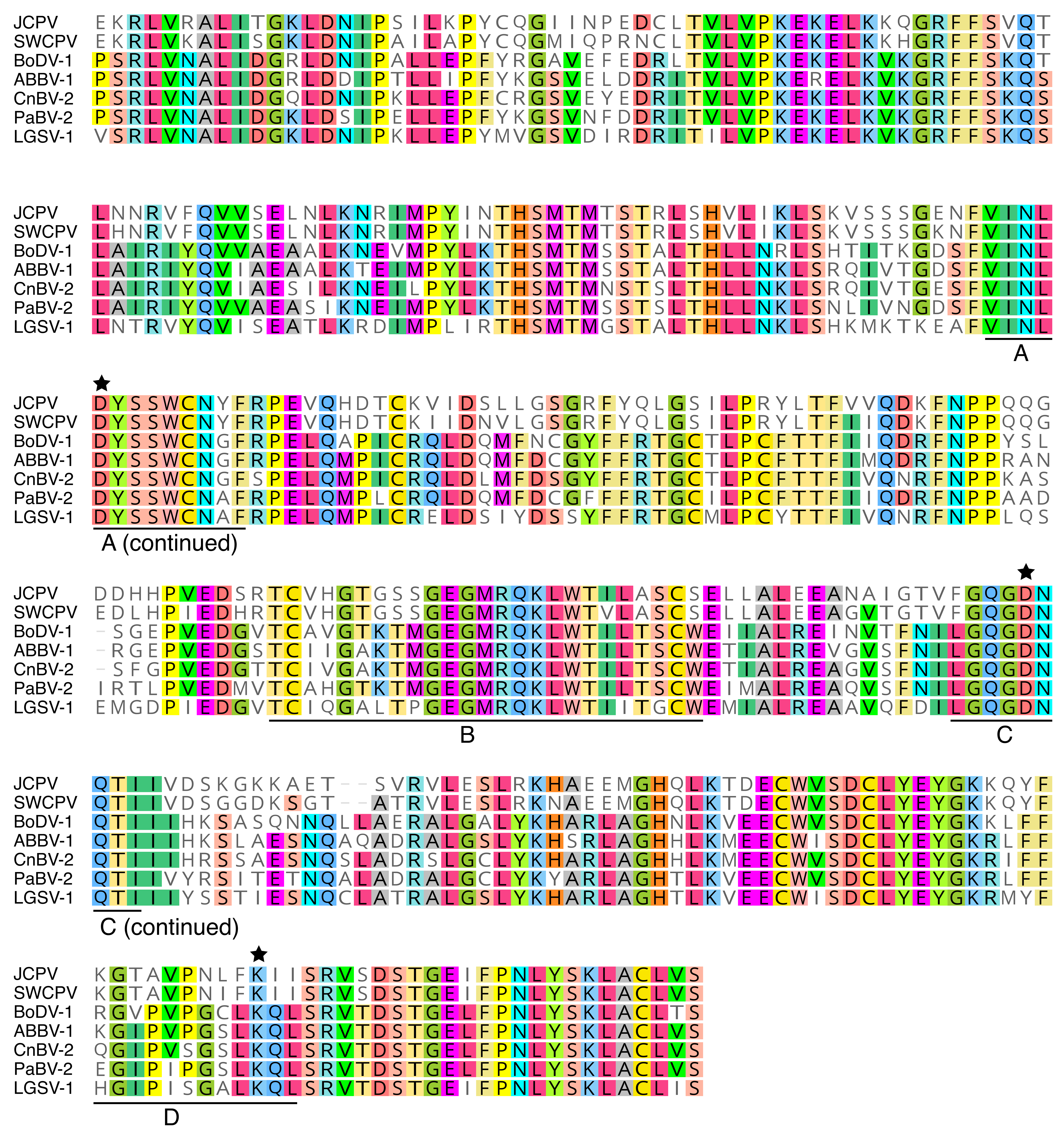

Supplement: S6 Fig — An alignment of the region of bornavirus L and EBLL sequences that includes the conserved A, B, C, and D motifs in Block III that were described in Poch et al. [53]. Residues that were found by Poch et al. [53] to be universally conserved are indicated by stars. Consensus residues shared by >50% of the sequences are highlighted. Sequence accessions: JCPV: MF135780; SWCPV: MF135781; BoDV-1: NC_001607.1; ABBV-1: NC_029642.1; CnBV-2: NC_027892.1; NC_028106.1; LGSV-1: NC_024778.1. Alignment corresponds to residues 337–645 of BoDV-1 L. (TIFF) [file ppat.1006881.s006.tiff]

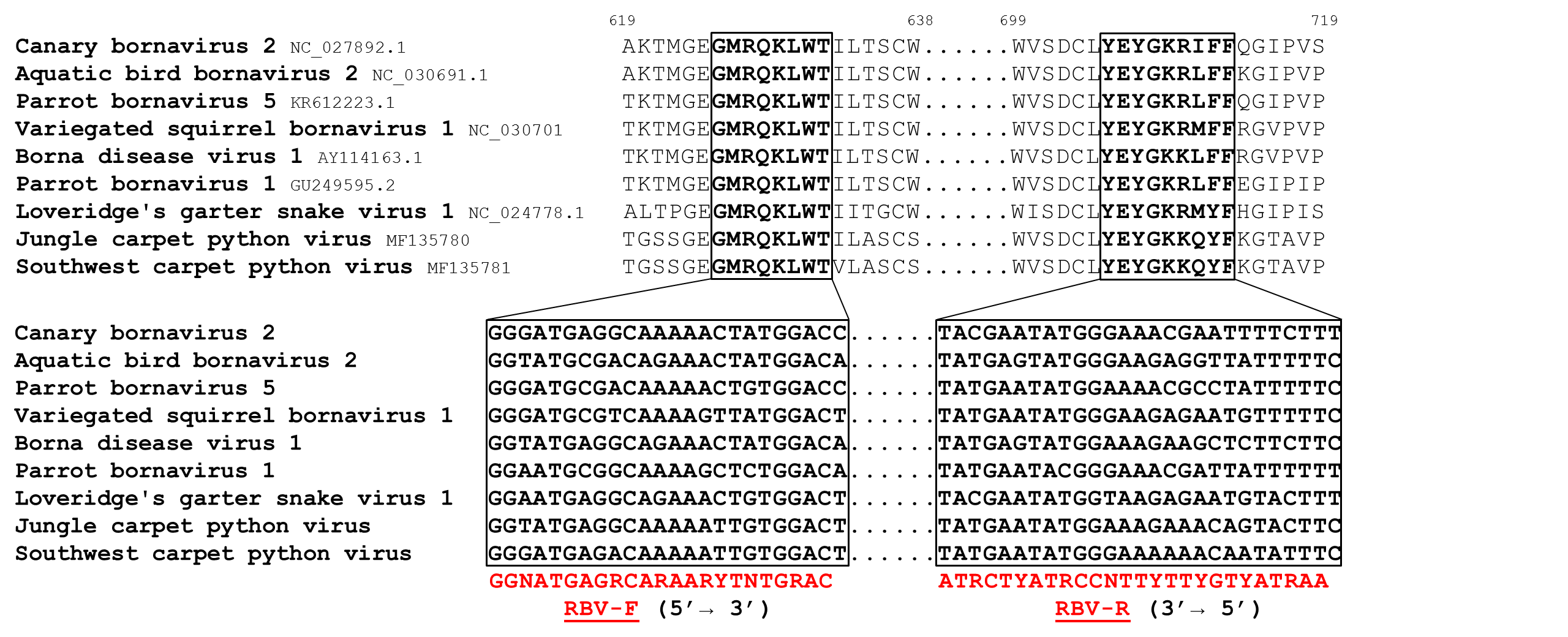

Supplement: S7 Fig — A multiple sequence alignment of the putative jungle carpet python virus and southwest carpet python virus L proteins with the cognate L protein sequences from seven of the eight species of the genus Bornavirus. This area of the genome has not been sequenced in a Passeriform 2 bornavirus. Each virus name is followed by its GenBank accession number and columns are labelled relative to the amino acid residue number of Borna disease virus-1 L protein (AAM68151). (TIFF) [file ppat.1006881.s007.tiff]

chr10:100000000-100000000

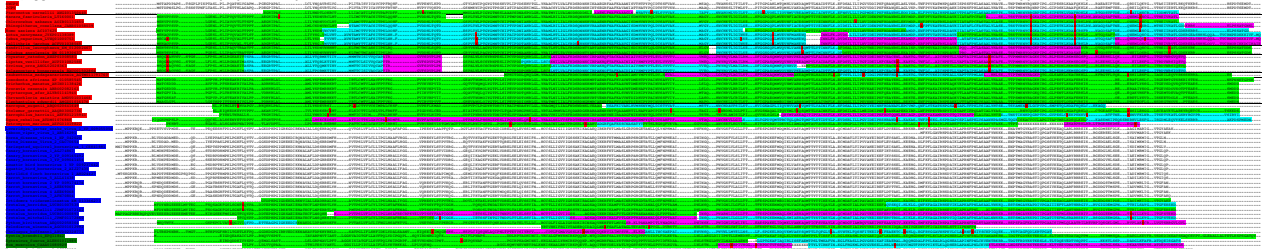

Supplement: S1 Appendix — EBLG, EBLL and EBLN nucleotide sequences in fasta format followed by amino acid sequences corresponding to frames 1–3. (ZIP) [file ppat.1006881.s008.zip › Borna N MAFFT alignment.pdf]
