# Supplementary material for: Divergent bornaviruses from Australian carpet pythons with neurological disease date the origin of extant Bornaviridae prior to the end-Cretaceous extinction
Source: PLoS Pathog. 2018 Feb 20;14(2):e1006881. doi: 10.1371/journal.ppat.1006881 (PMC5834213; doi:10.1371/journal.ppat.1006881)
Supplement: S1 Appendix — EBLG, EBLL and EBLN nucleotide sequences in fasta format followed by amino acid sequences corresponding to frames 1–3. (ZIP) [file ppat.1006881.s008.zip › Borna G MAFFT alignment.pdf]

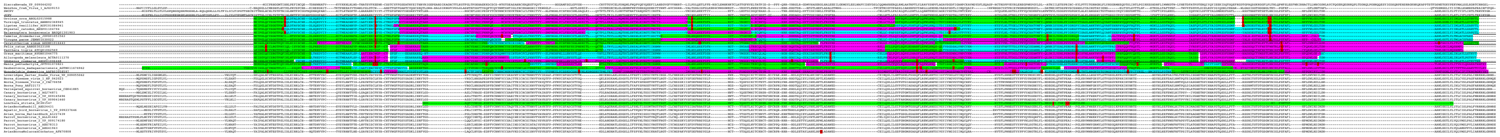
SierraNevada_YP_009044202
Wenzhou_Crab_Virus_1_AJG39153
SWCPV
JCPV
Orcinus_orca_ANOL02015988
Tursiops_truncatus_ABRN02368945
Lipotes_vexillifer_AUPI01084941
Physeter_catodon_AWZP01104794
Balaenoptera_bonaerensis_BAUQ01301903
Camelus_dromedarius_JDVD01051563
Vicugna_pacos_JEMW01030022
Ceratotherium_simum_AKZM01016443
Felis_catus_AANG03023108
Panthera_tigris_ATCQ01062569
Ursus_maritimus_AVOR01044775
Ailuropoda_melanoleuca_ACTA0111370
Odobenus_rosmarus_ANOP01006468
Manis_pentadactyla_JPTV01070821
Daubentonia_madagascariensis_AGTM011676962
Monodelphis_domestica_AAFR03058597
Loveridges_Garter_Snake_Virus_YP_009055062
Borna_disease_virus_1_NP_042023
Borna_Disease_Virus_2_CAC70658
Borna_Pony_AAF22173
Variegated_squirrel_bornavirus_CEK41885
Canary_bornavirus_1_AGJ74871
Canary_bornavirus_2_YP_009165496
Canary_bornavirus_3_YP_009041460
Lonchura_striata_DC281547
AvianBornaWaxbill_AHD24431
Aquatic_bird_bornavirus_1_YP_009237646
Avian_borna_MallardDuck_AIJ27439
Parrot_bornavirus_5_ALL41442
Parrot_bornavirus_2_YP_009174180
Parrot_bornavirus_1_AEG78313
Parrot_bornavirus_4_AEG41943
AvianBornaMoluccanCockatoo_AFN70808
