# Supplementary material for: Divergent bornaviruses from Australian carpet pythons with neurological disease date the origin of extant Bornaviridae prior to the end-Cretaceous extinction
Source: PLoS Pathog. 2018 Feb 20;14(2):e1006881. doi: 10.1371/journal.ppat.1006881 (PMC5834213; doi:10.1371/journal.ppat.1006881)
Supplement: S1 Appendix — EBLG, EBLL and EBLN nucleotide sequences in fasta format followed by amino acid sequences corresponding to frames 1–3. (ZIP) [file ppat.1006881.s008.zip › EBL L - frameshifts and AA sequences/Eptesicus_fuscus_ALEH01013293.doc]

>Eptesicus_fuscus_ALEH01013293

ACCTGACAAGTTTCCCAAGCAAGGGAGAATTGATCTATCAACTGAAATAA GTCGTGATTTGCTATCGTCCCAATTTGAGGTGGAGTCCTACAAGCTTGAATTATAGGGCCAAGACCTCTT GGAATTTATATGCGACGTCATGGCAATGTACCGTATGAACCAAGTCCTGAAGTCACCGCTTCTGGGAACT GAAGTCAAGATTGCTGTGTCTGGGAGTAAATTACCATCTCACCAAAGGCTTCTGAAGCTGATCCCTTCAC GAGAATGCCTGCAATCATCCAATGAATATCACAAGTTCTTCTTGAATCATTTTGTTGCACCTCATCTCTC CCATCATGACTTAACCCCAAGTGTCATGCTCATATGGAAAGCAGTTGCCAATACTTGGAGTGCACCCGAT GATTTGATAACCACTGTCTCTCACGAGCTTCCTTCACTTCTCCTGGACAATAAATTAGTGAGAACTTTAG AAAAACATTTGGCTTTGCAAGAAGGAGTTATAAAATCATCCTACTCTGAGCATGAAAGCATGACAATTAT GATTGGGAACCTAACCTGTCATTTTTCAAGATCACTTGTATTGGTTATTAGAGGGAGAGATTCCTGTCTT ATGACTTTCAACCACTACTTATCAGTTGCTGACACATACCGATCAAGAGTCTACTTAATTGTAGCTGGAC TAGTTGAAGACATGTTCCAAAAATGGCCTGCAAGTATAGCCATCAAGATAAATGAGTATATTGAGGACAT GGATACTGCAGCCAGTAAGCTCATGCATGATGATTATTTTGATGCTGTCAAATCTATATACCCATACTCA ATTGGAAAAGTACTTCAATATCATAATAAGGAACTCAAATCTAGCTTCCACTCAGTTACCGAGCCTTTAG TAGGACATCTGCCACTACGCTTGCTGGAGACCATCAATACTCTTGCAAAGGATTGTCCACAAATGGCGTT AGAACTATTTTCTGTGTCAAAGTGTTTATTCTTTCCAGAAATTGACATTGCCGAGGGAAGCAAGCAACAG TTTGCTAAAATGAGGAGAGAGACGGCTGATTCAACAAAATTCCATAAAATTGGAGGTGAGTTGCTCAATG TGTTCAGAGCTGAATACATAAGAGGTCACGTGAAAAAGCACTCAGCCTGGCCGTCTGTAACAATGCTTCC CAAATGTTCTAGAACTCTACGACAAGCCCAAATAGCAGGAGAATGGCCTAAATTTGGGACGTGGAAATAT GCTGATTTTGAGTATGTGCAATTGGAATATATTGGAAGAGGGCTTGAGTTTGAACCAGACTTAAGTGACA TTATAAAAGATACAGCAATTATTGAGGACTTAGATAGATGGTCATTTGAATACAATCAAGAAGCCTATCG AATCAAATTCCTTGAAAGACTTAAACATAGAGCTGATGAAAGAGGGGTTCAGCGATTAATTCTTGCCCTT CTTCGAGGTGATCTAGATGACATTAAAGGACAGCTCCAACCATTCATGACGGGAATGATCTCGAGGAAAG ACCTGATCACTGTATTAGTCCCCAAAGAGAAGGAGCTGAAGGTCAAAGGCAGGTATTTCCCAAAGCAATC TTGTAGGATTCGTCTTTATCAGGTGCTGGCAGAGTTAAATTTGAAGAAGTTTATTATGCCTTATTTACGG ATGCACTCTATGACTACCTCATCCACACAATTATCACACATACTTGACCGCATCTCCTCATTGCTCTCTA GAGGCAACAGATTCATAATCAATTTGGACTATGAGTCTTGGAGCAGCACCTTCAGGCCAGAGCTGCAAGA TCCCTTATGCAGAGAACTCGACAGAATGTTTGGCTCGGATTGTTTTTACCAAGTAGGATCATGGATGCCT TCCATTACAACATTCATAATGCAAGACAGATTCAACCCTCCACGGCAAGATGACAACGGATATCCATGTG ATGACATGGAGACATGCGCTCAAGGGACTACAACTATGGGAGAGGGAATGAGACAGAAGCTGTGGACAAT TCTAACAGCATGCTTAGAGCTTGTCACACTTGAAGCTGTTGGAATTCATGGTGAGGTCCTTGGTCAAGGT GATAATCAGACTATCTTAATTACACCCCCTCGCCACATGTCTCAAGAAGATGCAAGAGATGTTGTTATTA AACACTTGCAGCAAAGAAGTCATGAAGCGGGACTTGTTTTAAAGCCTGAAGAATGTTGGTCAAGTGATAT CCTTTATGAGTATGGAAAGAAATTATATTATGATGGTGTTCCTGTTCCAAATTTCCTCAAGATCTTCTCA AGGATTAGTGATAGTACAGGAGAAGTCTATCCAAATGTTTACTCTCGATTAGCGTGTTTAAGTTCAAGTT GTCTCTCCGCCTCTCAGTCAGATTATACCTCATGGCCATCTGTGGTGGGAGGCATCGTGGTCTATGTAAT AGAGATTGTGGTATTATTGCCTGAAGAAATATACAAGAATCACCAGCTTGTGGCAGCAATTGGACTTGTT GGCCCAATTCTAGGAGGCTTGCCTTCTCCAGCAACTTTACCATCAGTAATGCTGCGAGGTGTACCTGACC CATTGACATTCCAGTTATCTCTATTGAAGAGTGCAATTAGCATTGGAGTTTCTAAGAGTTGCATCCATCA AGTAGCAAAACTCCAAATTCCCAATGAACCAAGTGCATTAGCATTGTGTTCTGATCCAACCTGCCTAAAC ATTATACCATTAAGAAGACCTGAAATTGTGCTGAAAACCTGGATAGAAGAATCTTTATCAGAGATGATGG GATCATCTCGTGTACTTAGGCTCATGCAAATGGATATTACTGCCAAGGGGGAAGTCCTAGCCAAAGACTT GTTTAATATGGAGCCAAAGTATCCAAGATTAATGAGTTACCTGTTTACAAAGAGCAATGTTGTTTATGGG CTTTCAATGCTGGATAAATTTCAAAAGTCAAGCACAGTCATTGGAATAAGTCAAACAATGTCTATGAGAT CAATAATAGCACAAGAGAGTAGGACATTCAAAGATAGACTAATATCCAGCATCTTGGCACCAAACACCAC CAAATTAAACATATTAGATTACCTTCAAGCATGCTCAGTTGTTGCAGCTGATAAGTTGCGCCAAGACACA TGGGGAGTTAAGCTAGAAGGTGTCTCAATGCCCTTCATTGCAGAGCAATTTCACCTTCAGGAGACTGCTA CAAGTGAAGAGGTTGCCTGTTCAATAATATTTCGCCCTGATACAAAACTGTTTCCCCGTCAATGGACCAA AAGAGGATCAAGGCCATTATACATAGGTTCGAGAACCCATATCAAGGTAAATAGAGGAGCAATTACAGGT TTGCCGGAAGGAAAGATAGGTAAGATGGTTGAAGACCTAATCCTCACTTGTAATTGGATCAAATTGCGAG GGTCTGTTGGTGGCAACATGGCAAACTTGCTTCTCCAACTACTCAAAGAAAAGGGTGTCACTTCACCCAC TTTGCCTGTCATAATGGGAGGCACTCTAACACATAGATTGCCTACTGCATCAGATGACCGTGCAGGCTTG GCGGGGAATTTGAATGTGATCTCAACTCATATTAATTTTACCACTAATTATATGACCAACTTCACAAAGT CTCAGGAAGATTACACAATTCATTTTCAAGCTGCATTCTTGCATGGACTTAACTTGCTAGCATCAAAGCT CCACTATGGTACGTTGGAACCAATTACATACTACCTCACCACTAGTTGCAAAACATGCACTTCACTGATT GAGGAAGGAGGATTTGAGTTAACTCGACCGGCTGCTTACAAAGGAATTGATCTCAAAGACTTTCCCTCAA ATTACACTGTACCACCTTCTGAATGGATAGGGGATCCTGCTATAGCTGCAGCCTACAAGCTTGGATTTGA AATCTTTACATCTTTGCATCAAGAATCAAGAGGTTTAGCAACACTACTTGATTCCGATCAATCTCCTCAA AATCTTGAAAGACTCTCATTAAGTCACTTGTCTCTGCTTTCCTGTCATTTGTTGGTATGTTCATTTTGGT TTGCAGGCTCCTTGAAGAGAGGCATCTTACCTTCTGTTTGCCATCAACTCAAACTTGCATTATTTCTACC TCAATCTTCTCCAACTCTCAGGTGGGTGTGTGATGTTATATTCTCATTGGCAGATGGTAACCAAGTGTTT GATTTAGCTCACAAGGTAGGAGTGTTACAATCATGTGTAGTTAAAGGTACGTCTCCTAGAAATTCTCTAA TTGTTAACTTGCTACTTAGTGGCCTTGCTCAAGAGTGCCCTCGAGTAACCCAAATAATGCACAGACTCAA GTTTGATAAAATTCACAGATATTGTGAGAATTCACCTGATATAGATTCATTATTGGGAATGTTGAGACCA TTGACTACCAAAGTAAAAACTGAATGTAATGCAAAAGAAACACTTGGGTTCATAAGGAGTAGTGGATGTC AAATGCATACACAAGTCCACTCACTTCCAACCTCTGAGAATTGGCACCTTGCATACTTGTTGAAAGCTTT CATAGCACTGTATGATGTTCCTGTGATGGTGGTGTCTGGCAATACGCCTTATGATGTGATACTGGATTTA TGTCATGTAATTCCAGTGGTGGTGAATGCAGAGGGTGATCCGGGCATCATAGAAAAAGTTGAATTTGCAG ATATGTGTGGTGCAGTATCTAGAAATTTGAAATGTGAGCTATCCCTTTCAACCTTGATTTATGCCAATTA TTATTATGATCACTCTCAACTTATTAAAGGAAGTATTCACTACAAGGCGATTCCAGACCTGATCTTATCA CAAGGACCTTGTGAAACTACACAAACAATGGGTTTATTTTACGGGGACAAATTGATTGATCTTAGTGATG TGAGTTGCAAGCCTTATGATGTTGCTGATCTGTGTCCCTCGGTTTGTCCTGTTCTGGAGGCTATGTATAA GAGTGTAGTTGAGGTTGCCAATTCCTGCCTAGGAGCCTTGGAAGACTTTCCAGGTAAAGTGCTAGAGTCC AGGAGAGCAGAATCTGAATGGACTAGAACGGCAATTGTACGGCAAGAGGTATCCATATTAGTTCAAGAGT TGCATGAGTACTTGAATATTCTTCGGTTAGCACTGGGCTTACGGCATTATAAAACCAGGACTTATTACTT AAGGCTCAAGAAAGACATGAAGCATCTGTCCACGAGAAGACCTGGCAACCCTATACTCTGTAAAAGTTCA ATGGTATCTTTGAGAATTCAGCCAGATCCTTCCGTGTCTCTACATCATATTGCACACTTGCTCTAGGTAG CCCTACCGTGACATGGTAATATGATTAAAGAAAAACATATTAATAGGTCATTAACCCCCCTTTTTAAATT TGTTTATTGAGTATAGATCCATGTCTTTATGACTTATGTCTCATGTCTCATTCATTAGGTGCACTAAAAT GCCCCCCAGCCTGAGATCCACTTGCTATACTGACAGAAGCAACAATTTGCAAATAGGTTGTTTCGTGTGT ATGAGCTGCATATTAATC

>Frame1

TXQVSQARENXSINXNKSXFAIVPIXGGVLQAXIIGPRPLGIYMRRHGNVPYEPSPEVTASGNXSQDCCVWEXITISPKASEADPFTRMPAIIQXISQVLLESFCCTSSLPSXLNPKCHAHMESSCQYLECTRXFDNHCLSRASFTSPGQXISENFRKTFGFARRSYKIILLXAXKHDNYDWEPNLSFFKITCIGYXRERFLSYDFQPLLISCXHIPIKSLLNCSWTSXRHVPKMACKYSHQDKXVYXGHGYCSQXAHAXXLFXCCQIYIPILNWKSTSISXXGTQIXLPLSYRAFSRTSATTLAGDHQYSCKGLSTNGVRTIFCVKVFILSRNXHCRGKQATVCXNEERDGXFNKIPXNWRXVAQCVQSXIHKRSREKALSLAVCNNASQMFXNSTTSPNSRRMAXIWDVEICXFXVCAIGIYWKRAXVXTRLKXHYKRYSNYXGLRXMVIXIQSRSLSNQIPXKTXTXSXXKRGSAINSCPSSRXSRXHXRTAPTIHDGNDLEERPDHCISPQREGAEGQRQVFPKAILXDSSLSGAGRVKFEEVYYALFTDALYDYLIHTIITHTXPHLLIALXRQQIHNQFGLXVLEQHLQARAARSLMQRTRQNVWLGLFLPSRIMDAFHYNIHNARQIQPSTARXQRISMXXHGDMRSRDYNYGRGNETEAVDNSNSMLRACHTXSCWNSWXGPWSRXXSDYLNYTPSPHVSRRCKRCCYXTLAAKKSXSGTCFKAXRMLVKXYPLXVWKEIILXWCSCSKFPQDLLKDXXXYRRSLSKCLLSISVFKFKLSLRLSVRLYLMAICGGRHRGLCNRDCGIIAXRNIQESPACGSNWTCWPNSRRLAFSSNFTISNAARCTXPIDIPVISIEECNXHWSFXELHPSSSKTPNSQXTKCISIVFXSNLPKHYTIKKTXNCAENLDRRIFIRDDGIISCTXAHANGYYCQGGSPSQRLVXYGAKVSKINELPVYKEQCCLWAFNAGXISKVKHSHWNKSNNVYEINNSTREXDIQRXTNIQHLGTKHHQIKHIRLPSSMLSCCSXXVAPRHMGSXARRCLNALHCRAISPSGDCYKXRGCLFNNISPXYKTVSPSMDQKRIKAIIHRFENPYQGKXRSNYRFAGRKDRXDGXRPNPHLXLDQIARVCWWQHGKLASPTTQRKGCHFTHFACHNGRHSNTXIAYCIRXPCRLGGEFECDLNSYXFYHXLYDQLHKVSGRLHNSFSSCILAWTXLASIKAPLWYVGTNYILPHHXLQNMHFTDXGRRIXVNSTGCLQRNXSQRLSLKLHCTTFXMDRGSCYSCSLQAWIXNLYIFASRIKRFSNTTXFRSISSKSXKTLIKSLVSAFLSFVGMFILVCRLLEERHLTFCLPSTQTCIISTSIFSNSQVGVXCYILIGRWXPSVXFSSQGRSVTIMCSXRYVSXKFSNCXLATXWPCSRVPSSNPNNAQTQVXXNSQILXEFTXYRFIIGNVETIDYQSKNXMXCKRNTWVHKEXWMSNAYTSPLTSNLXELAPCILVESFHSTVXCSCDGGVWQYALXCDTGFMSCNSSGGECRGXSGHHRKSXICRYVWCSIXKFEMXAIPFNLDLCQLLLXSLSTYXRKYSLQGDSRPDLITRTLXNYTNNGFILRGQIDXSXXCELQALXCCXSVSLGLSCSGGYVXECSXGCQFLPRSLGRLSRXSARVQESRIXMDXNGNCTARGIHISSRVAXVLEYSSVSTGLTALXNQDLLLKAQERHEASVHEKTWQPYTLXKFNGIFENSARSFRVSTSYCTLALGSPTVTWXYDXRKTYXXVINPPFXICLLSIDPCLYDLCLMSHSLGALKCPPAXDPLAILTEATICKXVVSCVXAAYX

>Frame2

PDKFPKQGRIDLSTEISRDLLSSQFEVESYKLELXGQDLLEFICDVMAMYRMNQVLKSPLLGTEVKIAVSGSKLPSHQRLLKLIPSRECLQSSNEYHKFFLNHFVAPHLSHHDLTPSVMLIWKAVANTWSAPDDLITTVSHELPSLLLDNKLVRTLEKHLALQEGVIKSSYSEHESMTIMIGNLTCHFSRSLVLVIRGRDSCLMTFNHYLSVADTYRSRVYLIVAGLVEDMFQKWPASIAIKINEYIEDMDTAASKLMHDDYFDAVKSIYPYSIGKVLQYHNKELKSSFHSVTEPLVGHLPLRLLETINTLAKDCPQMALELFSVSKCLFFPEIDIAEGSKQQFAKMRRETADSTKFHKIGGELLNVFRAEYIRGHVKKHSAWPSVTMLPKCSRTLRQAQIAGEWPKFGTWKYADFEYVQLEYIGRGLEFEPDLSDIIKDTAIIEDLDRWSFEYNQEAYRIKFLERLKHRADERGVQRLILALLRGDLDDIKGQLQPFMTGMISRKDLITVLVPKEKELKVKGRYFPKQSCRIRLYQVLAELNLKKFIMPYLRMHSMTTSSTQLSHILDRISSLLSRGNRFIINLDYESWSSTFRPELQDPLCRELDRMFGSDCFYQVGSWMPSITTFIMQDRFNPPRQDDNGYPCDDMETCAQGTTTMGEGMRQKLWTILTACLELVTLEAVGIHGEVLGQGDNQTILITPPRHMSQEDARDVVIKHLQQRSHEAGLVLKPEECWSSDILYEYGKKLYYDGVPVPNFLKIFSRISDSTGEVYPNVYSRLACLSSSCLSASQSDYTSWPSVVGGIVVYVIEIVVLLPEEIYKNHQLVAAIGLVGPILGGLPSPATLPSVMLRGVPDPLTFQLSLLKSAISIGVSKSCIHQVAKLQIPNEPSALALCSDPTCLNIIPLRRPEIVLKTWIEESLSEMMGSSRVLRLMQMDITAKGEVLAKDLFNMEPKYPRLMSYLFTKSNVVYGLSMLDKFQKSSTVIGISQTMSMRSIIAQESRTFKDRLISSILAPNTTKLNILDYLQACSVVAADKLRQDTWGVKLEGVSMPFIAEQFHLQETATSEEVACSIIFRPDTKLFPRQWTKRGSRPLYIGSRTHIKVNRGAITGLPEGKIGKMVEDLILTCNWIKLRGSVGGNMANLLLQLLKEKGVTSPTLPVIMGGTLTHRLPTASDDRAGLAGNLNVISTHINFTTNYMTNFTKSQEDYTIHFQAAFLHGLNLLASKLHYGTLEPITYYLTTSCKTCTSLIEEGGFELTRPAAYKGIDLKDFPSNYTVPPSEWIGDPAIAAAYKLGFEIFTSLHQESRGLATLLDSDQSPQNLERLSLSHLSLLSCHLLVCSFWFAGSLKRGILPSVCHQLKLALFLPQSSPTLRWVCDVIFSLADGNQVFDLAHKVGVLQSCVVKGTSPRNSLIVNLLLSGLAQECPRVTQIMHRLKFDKIHRYCENSPDIDSLLGMLRPLTTKVKTECNAKETLGFIRSSGCQMHTQVHSLPTSENWHLAYLLKAFIALYDVPVMVVSGNTPYDVILDLCHVIPVVVNAEGDPGIIEKVEFADMCGAVSRNLKCELSLSTLIYANYYYDHSQLIKGSIHYKAIPDLILSQGPCETTQTMGLFYGDKLIDLSDVSCKPYDVADLCPSVCPVLEAMYKSVVEVANSCLGALEDFPGKVLESRRAESEWTRTAIVRQEVSILVQELHEYLNILRLALGLRHYKTRTYYLRLKKDMKHLSTRRPGNPILCKSSMVSLRIQPDPSVSLHHIAHLLXVALPXHGNMIKEKHINRSLTPLFKFVYXVXIHVFMTYVSCLIHXVHXNAPQPEIHLLYXQKQQFANRLFRVYELHIN

>Frame3

LTSFPSKGELIYQLKXVVICYRPNLRWSPTSLNYRAKTSWNLYATSWQCTVXTKSXSHRFWELKSRLLCLGVNYHLTKGFXSXSLHENACNHPMNITSSSXIILLHLISPIMTXPQVSCSYGKQLPILGVHPMIXXPLSLTSFLHFSWTINXXELXKNIWLCKKELXNHPTLSMKAXQLXLGTXPVIFQDHLYWLLEGEIPVLXLSTTTYQLLTHTDQESTXLXLDXLKTCSKNGLQVXPSRXMSILRTWILQPVSSCMMIILMLSNLYTHTQLEKYFNIIIRNSNLASTQLPSLXXDICHYACWRPSILLQRIVHKWRXNYFLCQSVYSFQKLTLPREASNSLLKXGERRLIQQNSIKLEVSCSMCSELNTXEVTXKSTQPGRLXQCFPNVLELYDKPKXQENGLNLGRGNMLILSMCNWNILEEGLSLNQTXVTLXKIQQLLRTXIDGHLNTIKKPIESNSLKDLNIELMKEGFSDXFLPFFEVIXMTLKDSSNHSXREXSRGKTXSLYXSPKRRSXRSKAGISQSNLVGFVFIRCWQSXIXRSLLCLIYGCTLXLPHPHNYHTYLTASPHCSLEATDSXSIWTMSLGAAPSGQSCKIPYAENSTECLARIVFTKXDHGCLPLQHSXCKTDSTLHGKMTTDIHVMTWRHALKGLQLWEREXDRSCGQFXQHAXSLSHLKLLEFMVRSLVKVIIRLSXLHPLATCLKKMQEMLLLNTCSKEVMKRDLFXSLKNVGQVISFMSMERNYIMMVFLFQISSRSSQGLVIVQEKSIQMFTLDXRVXVQVVSPPLSQIIPHGHLWWEASWSMXXRLWYYCLKKYTRITSLWQQLDLLAQFXEACLLQQLYHQXCCEVYLTHXHSSYLYXRVQLALEFLRVASIKXQNSKFPMNQVHXHCVLIQPAXTLYHXEDLKLCXKPGXKNLYQRXWDHLVYLGSCKWILLPRGKSXPKTCLIWSQSIQDXXVTCLQRAMLFMGFQCWINFKSQAQSLEXVKQCLXDQXXHKRVGHSKIDXYPASWHQTPPNXTYXITFKHAQLLQLISCAKTHGELSXKVSQCPSLQSNFTFRRLLQVKRLPVQXYFALIQNCFPVNGPKEDQGHYTXVREPISRXIEEQLQVCRKERXVRWLKTXSSLVIGSNCEGLLVATWQTCFSNYSKKRVSLHPLCLSXWEALXHIDCLLHQMTVQAWRGIXMXSQLILILPLIIXPTSQSLRKITQFIFKLHSCMDLTCXHQSSTMVRWNQLHTTSPLVAKHALHXLRKEDLSXLDRLLTKELISKTFPQITLYHLLNGXGILLXLQPTSLDLKSLHLCIKNQEVXQHYLIPINLLKILKDSHXVTCLCFPVICWYVHFGLQAPXREASYLLFAINSNLHYFYLNLLQLSGGCVMLYSHWQMVTKCLIXLTRXECYNHVXLKVRLLEILXLLTCYLVALLKSALEXPKXCTDSSLIKFTDIVRIHLIXIHYWECXDHXLPKXKLNVMQKKHLGSXGVVDVKCIHKSTHFQPLRIGTLHTCXKLSXHCMMFLXWWCLAIRLMMXYWIYVMXFQWWXMQRVIRASXKKLNLQICVVQYLEIXNVSYPFQPXFMPIIIMITLNLLKEVFTTRRFQTXSYHKDLVKLHKQWVYFTGTNXLILVMXVASLMMLLICVPRFVLFWRLCIRVXLRLPIPAXEPWKTFQVKCXSPGEQNLNGLERQLYGKRYPYXFKSCMSTXIFFGXHWAYGIIKPGLITXGSRKTXSICPREDLATLYSVKVQWYLXEFSQILPCLYIILHTCSRXPYRDMVIXLKKNILIGHXPPFLNLFIEYRSMSLXLMSHVSFIRCTKMPPSLRSTCYTDRSNNLQIGCFVCMSCILI

>Eptesicus_fuscus_ALEH01013293

VMAMYRMNQVLKSPLLGTEVKIAVSGSKLPSHQRLLKLIPSRECLQSSNEYHKFFLNHFVAPHLSHHDLTPSVMLIWKAVANTWSAPDDLITTVSHELPSLLLDNKLVRTLEKHLALQEGVIKSSYSEHESMTIMIGNLTCHFSRSLVLVIRGRDSCLMTFNHYLSVADTYRSRVYLIVAGLVEDMFQKWPASIAIKINEYIEDMDTAASKLMHDDYFDAVKSIYPYSIGKVLQYHNKELKSSFHSVTEPLVGHLPLRLLETINTLAKDCPQMALELFSVSKCLFFPEIDIAEGSKQQFAKMRRETADSTKFHKIGGELLNVFRAEYIRGHVKKHSAWPSVTMLPKCSRTLRQAQIAGEWPKFGTWKYADFEYVQLEYIGRGLEFEPDLSDIIKDTAIIEDLDRWSFEYNQEAYRIKFLERLKHRADERGVQRLILALLRGDLDDIKGQLQPFMTGMISRKDLITVLVPKEKELKVKGRYFPKQSCRIRLYQVLAELNLKKFIMPYLRMHSMTTSSTQLSHILDRISSLLSRGNRFIINLDYESWSSTFRPELQDPLCRELDRMFGSDCFYQVGSWMPSITTFIMQDRFNPPRQDDNGYPCDDMETCAQGTTTMGEGMRQKLWTILTACLELVTLEAVGIHGEVLGQGDNQTILITPPRHMSQEDARDVVIKHLQQRSHEAGLVLKPEECWSSDILYEYGKKLYYDGVPVPNFLKIFSRISDSTGEVYPNVYSRLACLSSSCLSASQSDYTSWPSVVGGIVVYVIEIVVLLPEEIYKNHQLVAAIGLVGPILGGLPSPATLPSVMLRGVPDPLTFQLSLLKSAISIGVSKSCIHQVAKLQIPNEPSALALCSDPTCLNIIPLRRPEIVLKTWIEESLSEMMGSSRVLRLMQMDITAKGEVLAKDLFNMEPKYPRLMSYLFTKSNVVYGLSMLDKFQKSSTVIGISQTMSMRSIIAQESRTFKDRLISSILAPNTTKLNILDYLQACSVVAADKLRQDTWGVKLEGVSMPFIAEQFHLQETATSEEVACSIIFRPDTKLFPRQWTKRGSRPLYIGSRTHIKVNRGAITGLPEGKIGKMVEDLILTCNWIKLRGSVGGNMANLLLQLLKEKGVTSPTLPVIMGGTLTHRLPTASDDRAGLAGNLNVISTHINFTTNYMTNFTKSQEDYTIHFQAAFLHGLNLLASKLHYGTLEPITYYLTTSCKTCTSLIEEGGFELTRPAAYKGIDLKDFPSNYTVPPSEWIGDPAIAAAYKLGFEIFTSLHQESRGLATLLDSDQSPQNLERLSLSHLSLLSCHLLVCSFWFAGSLKRGILPSVCHQLKLALFLPQSSPTLRWVCDVIFSLADGNQVFDLAHKVGVLQSCVVKGTSPRNSLIVNLLLSGLAQECPRVTQIMHRLKFDKIHRYCENSPDIDSLLGMLRPLTTKVKTECNAKETLGFIRSSGCQMHTQVHSLPTSENWHLAYLLKAFIALYDVPVMVVSGNTPYDVILDLCHVIPVVVNAEGDPGIIEKVEFADMCGAVSRNLKCELSLSTLIYANYYYDHSQLIKGSIHYKAIPDLILSQGPCETTQTMGLFYGDKLIDLSDVSCKPYDVADLCPSVCPVLEAMYKSVVEVANSCLGALEDFPGKVLESRRAESEWTRTAIVRQEVSILVQELHEYLNILRLALGLRHYKTRTYYLRLKKDMKHLSTRRPGNPILCKSSMVSLRIQPDPSVS
