# Supplementary material for: Divergent bornaviruses from Australian carpet pythons with neurological disease date the origin of extant Bornaviridae prior to the end-Cretaceous extinction
Source: PLoS Pathog. 2018 Feb 20;14(2):e1006881. doi: 10.1371/journal.ppat.1006881 (PMC5834213; doi:10.1371/journal.ppat.1006881)
Supplement: S1 Appendix — EBLG, EBLL and EBLN nucleotide sequences in fasta format followed by amino acid sequences corresponding to frames 1–3. (ZIP) [file ppat.1006881.s008.zip › EBL N - frameshifts and AA sequences/Colobus_angolensis_XM_011930637.doc]

>Colobus_angolensis_XM_011930637

TTTAATGGCTTAAAAGGGTTGGCATGGCCTCCAAAAGTAACTAACAGACCCTGCCAAGGGAAACCAAGAGAGGTCCCCTCAAGATATGGAGCACACATCAGTCTCTATATCCTTTGGGGAAAGCCTCCTACTTATTCCAGCACAAGTCACACGTGCCACAACCCCTACAGCAGAGGGACATATTCCAGCCTTGTTAATACTGTACAGTGATAATGTGTGGCACTACCATACCAAATTAACAATGTGCACAATGATTGCGTCCAGCATCATACCATCACTACGGCAACCTGTTGATGATCATCTAAGCCAACCTGATGGAAGGTATGTAAGCTGGTTACCAGCGGGATATGGAACAGACACATGCAAAATTCCAACAATCCCAGATAATGCAGCTCACAGGCTTGGATTATATCATGCAATATTACATACTCTTACTGCTGTTTACAGAGATTCGAGAGATAACACCTATGCAAGGACTGTTGAAAACAGATTTAATACATTCAAAGCTGCTGCAGGAATCAGTGACATACACATCAGTTGCCTACAGCTTGGTGTTTCTGCCTATGTTGCAAAAAAGTTGGGATACCCTGGTGTCATAAAACCCTGATTGGAATACTGTCTGAAAAAGTTGGAGGATCTGGACAACCACTAATGGTTCAGGTTCATCTAGTGGCCCATGAGTAACAGATGACAACACTTGTGATGATAAATAACTTCCTTAAGGATTTTGAGGCCAGGTGCGGTGGCTCATGCCTGTAATCCCACACTTTGGGAGGCTGAGGCGGGTGAATCACCTGAGGCCAGGAGTTTCAGACCAGCCTGGCCAACATGGTGAAACCTCGTCTCTATTAAAATACAAAAATTAGCTGGGCATGGTGGTGCATGCCTATAATCCTAGCTACTCGGGAGGCTGAGGCAGGAGAATCATTTGAATCCAGGAGGCAGGGGTTGCAATGAGATGAGATTGGGCCACTGCACTCCAGCCTGGGTGACAGAATGAGACTTTGTCTCAAAAAAAAAAAAAAATTACGACTCCACACTTATTTTGATTCCAGGAGTAACTGATGAAATCCCCAAATTCAGAGATGCTTGGAGATCCCTTGAGAAAGCCATCAGTCCTGTGTGGTTTCCCTCTGTGAAGGTAATTTGCCATCCAGATGCTGTGAAAATCACCACGACAGCCTTGCCTTGCCTCCACAGCACTGTACCATGCCTCGGAAACAAGCTCCACCATGAGACATTACCAGCAAGGAAAACCAATTCCAGGAGGAATTCCTGAAACTAAGCTCAAGGATGCATCTGCTGAAAGCTCAGATAGGTTGGCCAATCAACATTCACAGATGAGCAATGGGAAATTCTGCAATCACGTGGAGTGACCCATGAAATTGTCTAGGCAACTCTTGGAGACAAAAAAAAGAGAAGAGAATCACCATCATCTGCAACAGGTGACAGTCCATAGGGACCCATGGATTTAAAAAAAAATTGAACCTCACAAAGAGATTCAACAGTCAGCTAATGAGTCAAAGGCCACAACCAGTAT

>Frame1

FNGLKGLAWPPKVTNRPCQGKPREVPSRYGAHISLYILWGKPPTYSSTSHTCHNPYSRGTYSSLVNTVQXXCVALPYQINNVHNDCVQHHTITTATCXXSSKPTXWKVCKLVTSGIWNRHMQNSNNPRXCSSQAWIISCNITYSYCCLQRFERXHLCKDCXKQIXYIQSCCRNQXHTHQLPTAWCFCLCCKKVGIPWCHKTLIGILSEKVGGSGQPLMVQVHLVAHEXQMTTLVMINNFLKDFEARCGGSCLXSHTLGGXGGXITXGQEFQTSLANMVKPRLYXNTKISWAWWCMPIILATREAEAGESFESRRQGLQXDEIGPLHSSLGDRMRLCLKKKKKLRLHTYFDSRSNXXNPQIQRCLEIPXESHQSCVVSLCEGNLPSRCCENHHDSLALPPQHCTMPRKQAPPXDITSKENQFQEEFLKLSSRMHLLKAQIGWPINIHRXAMGNSAITWSDPXNCLGNSWRQKKEKRITIICNRXQSIGTHGFKKKLNLTKRFNSQLMSQRPQPV

>Frame2

LMAXKGWHGLQKXLTDPAKGNQERSPQDMEHTSVSISFGESLLLIPAQVTRATTPTAEGHIPALLILYSDNVWHYHTKLTMCTMIASSIIPSLRQPVDDHLSQPDGRYVSWLPAGYGTDTCKIPTIPDNAAHRLGLYHAILHTLTAVYRDSRDNTYARTVENRFNTFKAAAGISDIHISCLQLGVSAYVAKKLGYPGVIKPXLEYCLKKLEDLDNHXWFRFIXWPMSNRXQHLXXXITSLRILRPGAVAHACNPTLWEAEAGESPEARSFRPAWPTWXNLVSIKIQKLAGHGGACLXSXLLGRLRQENHLNPGGRGCNEMRLGHCTPAWVTEXDFVSKKKKNYDSTLILIPGVTDEIPKFRDAWRSLEKAISPVWFPSVKVICHPDAVKITTTALPCLHSTVPCLGNKLHHETLPARKTNSRRNSXNXAQGCICXKLRXVGQSTFTDEQWEILQSRGVTHEIVXATLGDKKKRRESPSSATGDSPXGPMDLKKNXTSQRDSTVSXXVKGHNQY

>Frame3

XWLKRVGMASKSNXQTLPRETKRGPLKIWSTHQSLYPLGKASYLFQHKSHVPQPLQQRDIFQPCXYCTVIMCGTTIPNXQCAQXLRPASYHHYGNLLMIIXANLMEGMXAGYQRDMEQTHAKFQQSQIMQLTGLDYIMQYYILLLLFTEIREITPMQGLLKTDLIHSKLLQESVTYTSVAYSLVFLPMLQKSWDTLVSXNPDWNTVXKSWRIWTTTNGSGSSSGPXVTDDNTCDDKXLPXGFXGQVRWLMPVIPHFGRLRRVNHLRPGVSDQPGQHGETSSLLKYKNXLGMVVHAYNPSYSGGXGRRIIXIQEAGVAMRXDWATALQPGXQNETLSQKKKKITTPHLFXFQEXLMKSPNSEMLGDPLRKPSVLCGFPLXRXFAIQMLXKSPRQPCLASTALYHASETSSTMRHYQQGKPIPGGIPETKLKDASAESSDRLANQHSQMSNGKFCNHVEXPMKLSRQLLETKKREENHHHLQQVTVHRDPWIXKKIEPHKEIQQSANESKATTS

>Colobus_angolensis_XM_011930637

MEHTSVSISFGESLLLIPAQVTRATTPTAEGHIPALLILYSDNVWHYHTKLTMCTMIASSIIPSLRQPVDDHLSQPDGRYVSWLPAGYGTDTCKIPTIPDNAAHRLGLYHAILHTLTAVYRDSRDNTYARTVENRFNTFKAAAGISDIHISCLQLGVSAYVAKKLGYPWCHKTLIGILSEKVGGSGQPLMVQVHLVAHEXQMTTLVMINNFLKDFDSTLILIPGVTDEIPKFRDAWRSLEKAISPVWFPSVKVICHPDAVKIPRQPCLASTALYHASETSSTMRHYQQGKPIPGGIPETKLKDASAEKLRXVGQSTFTDEQWEILQSRGVTHEIVXATLGDKKKRRESPSSATGDS

>Frame1

KAQIGWPIN

>Frame2

KLRXVGQST

>Frame3

SSDRLANQH

>RBV3

KLKRAEAEI

Colobus angolensis palliatus solute carrier family 4, sodium bicarbonate cotransporter, member 8 (SLC4A8), transcript variant X1, mRNA

NCBI Reference Sequence: XM_011930637.1
