# Supplementary material for: Divergent bornaviruses from Australian carpet pythons with neurological disease date the origin of extant Bornaviridae prior to the end-Cretaceous extinction
Source: PLoS Pathog. 2018 Feb 20;14(2):e1006881. doi: 10.1371/journal.ppat.1006881 (PMC5834213; doi:10.1371/journal.ppat.1006881)
Supplement: S1 Appendix — EBLG, EBLL and EBLN nucleotide sequences in fasta format followed by amino acid sequences corresponding to frames 1–3. (ZIP) [file ppat.1006881.s008.zip › EBL N - frameshifts and AA sequences/Crotalus_horridus_LVCR01007535.doc]

>Crotalus_horridus_LVCR01007535

ATTAGTGGAAGAAGAAATAAATAAGATTTTGATAAAATTAAAAATGTAGAAGATACTTGGCAGGGATATTTGAATTGAAAATGTTTTTATTTAAAGTTAATTTGATTATGGTATTGTAAACTAACGAAGCTTGAAAACAGGTTCAGGTTGAATAAATTTAAGCTAGAATTGGATATTTTAAATATATTATGCTATTTGAGGGGAAAAGAGAGGGAACACTTGATGTATTGATTGTACAATGTATATACGTTTTGTTTTATGTTTGATTACAATGAAGGGGAGGAGGAGGAGTTGATGTACACAGAAGGCTTTAGCCAATCTGCAGGAAAATTTGGATCACTTTCTGGAAACTTCTGACAGTATCCCGCTGATGACACAGAGCCACACCCAGGCATAAGGAAGAAGGCCAAAATTGAACATGCCACTAAGTGTTTTCTCAACCCTGAACGCAGAACACTGTATCATCCAGTGACTCCAAGCCTTGTGTTTTTATGCTATCTGATTCCTGGATTATGGACAGCTTTTAAGAATGCTGGAGTTCCTACTAGTTCTTACTTTACTCAACCGATTGGTGGGCATGTTCAAAGTCAGTCTCTCACAAATGATGCAATTGAAGATGTTCCACTTACTGCACTCGAACTGTCAAGTGTGATAGCCCATTGCACCACTCTGCTAATCAGTATAGTCCTTGGATCATCCACCAAAATTGCAGCTGGGTCCGCCCAAATCAAGAAAAGAATTAAAGCAATGTTTGTTACGCTTGGAAAACCAACCCATGCCGACATAGCCAATGCACTACAACTCTTCCCACCTACAGCTGCGATGGATTGGCTCAACTCCCAACCATGGATTGGAAATCTCATACTTGGTTTTCTCGACATTACATTCGAAACACCTGGAAAGGAAATGATGGACCAAGTTCATTTAGTTGCAGGTTATGCTCAAATGACAACATATTCAACCATCCAGGCTTATTTGGAACAATGTATGGATGGAACCATAGCCTTACCTGCTGTTGTCAAAGAAATTGAAGAATTTGCCAAGTATCAAAACAAATCCATCTCAAACTTGGACAATGGTTTAAATTTGCGGGTGCCATAAGACATTCTGAGGTAATCAAGCTTGCTCCAAAGGCATTTCCAAATCTCGCCAGTGCCGCCCTTTACTGGAGTAAACAAGAAAGTACATCAATGGGTGCCTACAAAGCGCCGGATATAGTACCAGGTGCGATTATCATTGAGTTTACTCTAGCACGCACCCATAGAAGAGGAATAGTGAGAGTGGGCATTGGAGATGAGATGGGCCCAAACACTACACGTATTCTCTCTGCGTATGGCATAACGGGATTTGAGAAAAGTAAAGATTGAACCAATTCCTTAAAAAAAAAATAAGGCTGTAGTATTATTAACTTTTTCATCTTCTTTACTACCAGGTCTTATTGGTATTACATTATGATTATTATTACTCTGTTGCTTTGCATTGAGAGCTTCTGCACCAGAGATAAATTCCTTGTGTATCTAATCATCTTGACCAAATAAAGTATTCTGTTCTATTCTGTTCTGTTCTATTCTATTTTCTATTCATACATCTGGCAATCAATCATATCAATGCAATAGTGGGACAATCTTCCTTAGTCCAGGTCCACATCTTTGCCCATAATTGCTCTATAGAAAGAATGCAAAAAAAAAGATTATATGAGTGATAAGAAAAATAAAAAGAAAAGGAAAATATTACCAAAGACCTTCTTTGATCTGGCACATGAACTTAACC

>Frame1

ISGRRNKXDFDKIKNVEDTWQGYLNXKCFYLKLIXLWYCKLTKLENRFRLNKFKLELDILNILCYLRGKEREHLMYXLYNVYTFCFMFDYNEGEEEELMYTEGFSQSAGKFGSLSGNFXQYPADDTEPHPGIRKKAKIEHATKCFLNPERRTLYHPVTPSLVFLCYLIPGLWTAFKNAGVPTSSYFTQPIGGHVQSQSLTNDAIEDVPLTALELSSVIAHCTTLLISIVLGSSTKIAAGSAQIKKRIKAMFVTLGKPTHADIANALQLFPPTAAMDWLNSQPWIGNLILGFLDITFETPGKEMMDQVHLVAGYAQMTTYSTIQAYLEQCMDGTIALPAVVKEIEEFAKYQNKSISNLDNGLNLRVPXDILRXSSLLQRHFQISPVPPFTGVNKKVHQWVPTKRRIXYQVRLSLSLLXHAPIEEEXXEWALEMRWAQTLHVFSLRMAXRDLRKVKIEPIPXKKNKAVVLLTFSSSLLPGLIGITLXLLLLCCFALRASAPEINSLCIXSSXPNKVFCSILFCSILFSIHTSGNQSYQCNSGTIFLSPGPHLCPXLLYRKNAKKKIIXVIRKIKRKGKYYQRPSLIWHMNLT

>Frame2

LVEEEINKILIKLKMXKILGRDIXIENVFIXSXFDYGIVNXRSLKTGSGXINLSXNWIFXIYYAIXGEKRGNTXCIDCTMYIRFVLCLITMKGRRRSXCTQKALANLQENLDHFLETSDSIPLMTQSHTQAXGRRPKLNMPLSVFSTLNAEHCIIQXLQALCFYAIXFLDYGQLLRMLEFLLVLTLLNRLVGMFKVSLSQMMQLKMFHLLHSNCQVXXPIAPLCXSVXSLDHPPKLQLGPPKSRKELKQCLLRLENQPMPTXPMHYNSSHLQLRWIGSTPNHGLEISYLVFSTLHSKHLERKXWTKFIXLQVMLKXQHIQPSRLIWNNVWMEPXPYLLLSKKLKNLPSIKTNPSQTWTMVXICGCHKTFXGNQACSKGISKSRQCRPLLEXTRKYINGCLQSAGYSTRCDYHXVYSSTHPXKRNSESGHWRXDGPKHYTYSLCVWHNGIXEKXRLNQFLKKKIRLXYYXLFHLLYYQVLLVLHYDYYYSVALHXELLHQRXIPCVSNHLDQIKYSVLFCSVLFYFLFIHLAINHINAIVGQSSLVQVHIFAHNCSIERMQKKRLYEXXEKXKEKENITKDLLXSGTXTX

>Frame3

XWKKKXIRFXXNXKCRRYLAGIFELKMFLFKVNLIMVLXTNEAXKQVQVEXIXARIGYFKYIMLFEGKREGTLDVLIVQCIYVLFYVXLQXRGGGGVDVHRRLXPICRKIWITFWKLLTVSRXXHRATPRHKEEGQNXTCHXVFSQPXTQNTVSSSDSKPCVFMLSDSWIMDSFXECWSSYXFLLYSTDWWACSKSVSHKXCNXRCSTYCTRTVKCDSPLHHSANQYSPWIIHQNCSWVRPNQEKNXSNVCYAWKTNPCRHSQCTTTLPTYSCDGLAQLPTMDWKSHTWFSRHYIRNTWKGNDGPSSFSCRLCSNDNIFNHPGLFGTMYGWNHSLTCCCQRNXRICQVSKQIHLKLGQWFKFAGAIRHSEVIKLAPKAFPNLASAALYWSKQESTSMGAYKAPDIVPGAIIIEFTLARTHRRGIVRVGIGDEMGPNTTRILSAYGITGFEKSKDXTNSLKKKXGCSIINFFIFFTTRSYWYYIMIIITLLLCIESFCTRDKFLVYLIILTKXSILFYSVLFYSIFYSYIWQSIISMQXWDNLPXSRSTSLPIIALXKECKKKDYMSDKKNKKKRKILPKTFFDLAHELN

>Crotalus_horridus_LVCR01007535

MFDYNEGEEEELMYTEGFSQSAGKFGSLSGNFXQYPADDTEPHPGIRKKAKIEHATKCFLNPERRTLYHPVTPSLVFLCYLIPGLWTAFKNAGVPTSSYFTQPIGGHVQSQSLTNDAIEDVPLTALELSSVIAHCTTLLISIVLGSSTKIAAGSAQIKKRIKAMFVTLGKPTHADIANALQLFPPTAAMDWLNSQPWIGNLILGFLDITFETPGKEMMDQVHLVAGYAQMTTYSTIQAYLEQCMDGTIALPAVVKEIEEFAQVSKQIHLKLGQWFKFAGAIRHSEVIKLAPKAFPNLASAALYWSKQESTSMGAYKAPDIVPGAIIIEFTLARTHRRGIVRVGIGDEMGPNTTRILSAYGITGFEKSKD
