# Supplementary material for: Divergent bornaviruses from Australian carpet pythons with neurological disease date the origin of extant Bornaviridae prior to the end-Cretaceous extinction
Source: PLoS Pathog. 2018 Feb 20;14(2):e1006881. doi: 10.1371/journal.ppat.1006881 (PMC5834213; doi:10.1371/journal.ppat.1006881)
Supplement: S1 Appendix — EBLG, EBLL and EBLN nucleotide sequences in fasta format followed by amino acid sequences corresponding to frames 1–3. (ZIP) [file ppat.1006881.s008.zip › EBL N - frameshifts and AA sequences/Mus_musculus_CAAA01080929.doc]

>Mus_musculus_CAAA01080929

AGCTGTGTTCCCTGCCAGATCCTCCAGACAAATTCTCTAAAAGTAGTGTCGGTGCCAGGACTGCTTGACCAAGCAGGTCACCTCAGCATGTAGGGGATGATGACTACTTCAGGGCTAACCTCTGCTGCGTAAAGGTGCTGTCTGTGATAGCCCCAATTAATAACATGGAGTCAATGTCTCAAGTGTACACAAAGCACAGGACCAAGGCATCCCAGATGGAATTGACAACAACCCATTGTACCAAGAACCCATTGTGCTCCCCTATCTAGACTTAGTGGTGCAGCCAGCAGCTATCACACATCTGACTGAGTCAATAGTAGCAAAAACTCTGGGTGGAGGTATATTATTGCCAATAATTTGTCTCTTGGCAATTTTTCTGTCTTTATATAAGACAAGAAAAAAGGCCTTGCTTCCACTTGTACAAGAGAACCAAGCTGGAGGGCTGAACCAAATGGTTAATGAAGTTAATGAGACACCTGCACTGAAGAATAAAATCTCAAAGGCTGCATTGCGCCGTGTCATTAGAAAACCTCAAGAGGTCACAGCTCAGCAAATAACCAGAAGAGTCGATGAACTATATGCAACCCTCTATCAAGGGTCGACTTCTCCAGTTGTTCATCTCGGTCCTGTAGTAGATTTGAAAGGTGTGGCTCTGTGGTTGTCCACCAATGATTTGGGTACTCTCTTCCTAAAGGCCTATAGTCCATGGAGGCTGATGTAGACCAGACTGCACAGTGTTTAGTAGTCAACTTAAACTAAGTGAGCCACCTATGCAAAGATGACAACAGAGGACAATGTAGGACAACTTATCTAACAATCAGAAACTTCATCAATAAAGGCCTATTTCATGGCAAGATGTTACCTGGAGTTGGTGTTGAATCAGAGCTTTTAAGAAGGTTGTGGTAAAGCTCAGATGTCTGCATGGGGACATTCCCCCCCCCCCCCTATATCAAAACTTTAAAGTTGCAGGACCATGAAAGGCTTGCTCCTGCCAACCTTCCAAACTTGTGCAAGGTCGAAAAACTCTTAGCAAGAGGAAACTCAATGGATCATTCAAAGATTGTAAGTCAACCAAAGAAAGCCTATCCAAGCTCACCTATCATGAGGTTGATTGGGCAGTAAATAAGCCACCAAAGAGAGGAAGAGGGCTCAGCAGTGCTGACAAAGCAGCAGTGAGAGAGGTCTCCAGCGCTACAGATGAGGACGTCGATAACAGCAAGGGCTCCAGAACAATAAGAAGCCATCAGTGTTAATGCCATCCTGGGTGTTATCCAGAAACAGTCAAAAGGGTTGTAGCTTTAGGAAGGTAGAGAACCATTGCCCTAAAGCTTTCTGTAGCCACTGATATTCCCAGTGGTATATGCAGTGGGTCTTGTTGGGTATCGTTAGTGATCCTAGGGCTCTGCAGTGCTCTGGTGTTGGGCAGGGCCGTACACTGGAGCCACCTATCACTGCTGTGTATGTGTCTGCTGGGCTGTTCCATGTGCCCGTGTTGATGGTTGGCAGGGTGAGCTGCTCTCATCCTAACATGTCTAGAGCACCAAGTGAAAGGATTGTTATCACTTGGCATTTAGTGCTTTGTACATGTCACTGAACTCAGGTGATCTTCAAGAGAAAAATTGGAAATCTTTATTTCTGATTGGAGCTCAGCTGAGACCAAGGCAGCAGGAGTAACCATGGAGCTGGTTTACTATTCAGTCAGCATTGACTGGGATACAGAACACACATTTCAGCATAAGACAAAACAGAATTATAGATGTCAGGTTATGGAGGGTTGTGGGTCCCTCATCTGCTCTGCCACAGGACATGGCCACTTGGAGAGGCAAGATGTGGGGAGTTTGACTGCACTTACCCCATGCCCCATGGATGGGTGTGGGCTGTAGGGAAGCCCCAGGCACCACTCAGGGGATGGGAAAGCACAGTCTTAGGCATGGGAAAGCCGGAACTGGTTCGGGGGTCTCTGGGCCTGCGATGAGGCTGGGGCCATGGGGTTCTCATGCTCTTGGACATCTGGGCACCACTGAGAGTCGAGGAAGATCTGAGAATGGATTTTGGGCTTGTGGGCTGGGGACAGCCTGTAGTCCAGAGTGGTGGGAGGGAGGTGGGGAGAGGAAGGAAACTCTGGCTTGTCCCAGCAGGGAAAGTCCTTGGCTTGACAGGCATCAGGCTTGGTGGTGATTGTGGCTGGGAGCACAGAGAGGCCTTTTACAGGAGGCTAGGCTGCAGCTCTTTAGGCAAAGGACTTCTCCAAGTCTCCACACATGAATCCCAATGAAAGAGACAGTCTGTGGTTCCAAACCATTTATTATCATGGTGGAAAGTGGATGCATAAAACCATACCCCACATCTCAGGGTGGGCCTGAGATTAAATACCTTTTGCAGGGAGGAATGTCTG

>Frame1

SCVPCQILQTNSLKVVSVPGLLDQAGHLSMXGMMTTSGLTSAAXRCCLXXPQLITWSQCLKCTQSTGPRHPRWNXQQPIVPRTHCAPLSRLSGAASSYHTSDXVNSSKNSGWRYIIANNLSLGNFSVFIXDKKKGLASTCTREPSWRAEPNGXXSXXDTCTEEXNLKGCIAPCHXKTSRGHSSANNQKSRXTICNPLSRVDFSSCSSRSCSRFERCGSVVVHQXFGYSLPKGLXSMEADVDQTAQCLVVNLNXVSHLCKDDNRGQCRTTYLTIRNFINKGLFHGKMLPGVGVESELLRRLWXSSDVCMGTFPPPPYIKTLKLQDHERLAPANLPNLCKVEKLLARGNSMDHSKIVSQPKKAYPSSPIMRLIGQXISHQREEEGSAVLTKQQXERSPALQMRTSITARAPEQXEAISVNAILGVIQKQSKGLXLXEGREPLPXSFLXPLIFPVVYAVGLVGYRXXSXGSAVLWCWAGPYTGATYHCCVCVCWAVPCARVDGWQGELLSSXHVXSTKXKDCYHLAFSALYMSLNSGDLQEKNWKSLFLIGAQLRPRQQEXPWSWFTIQSALTGIQNTHFSIRQNRIIDVRLWRVVGPSSALPQDMATWRGKMWGVXLHLPHAPWMGVGCREAPGTTQGMGKHSLRHGKAGTGSGVSGPAMRLGPWGSHALGHLGTTESRGRSENGFWACGLGTACSPEWWEGGGERKETLACPSRESPWLDRHQAWWXLWLGAQRGLLQEARLQLFRQRTSPSLHTXIPMKETVCGSKPFIIMVESGCIKPYPTSQGGPEIKYLLQGGMS

>Frame2

AVFPARSSRQILXKXCRCQDCLTKQVTSACRGXXLLQGXPLLRKGAVCDSPNXXHGVNVSSVHKAQDQGIPDGIDNNPLYQEPIVLPYLDLVVQPAAITHLTESIVAKTLGGGILLPIICLLAIFLSLYKTRKKALLPLVQENQAGGLNQMVNEVNETPALKNKISKAALRRVIRKPQEVTAQQITRRVDELYATLYQGSTSPVVHLGPVVDLKGVALWLSTNDLGTLFLKAYSPWRLMXTRLHSVXXSTXTKXATYAKMTTEDNVGQLIXQSETSSIKAYFMARCYLELVLNQSFXEGCGKAQMSAWGHSPPPPISKLXSCRTMKGLLLPTFQTCARSKNSXQEETQWIIQRLXVNQRKPIQAHLSXGXLGSKXATKERKRAQQCXQSSSERGLQRYRXGRRXQQGLQNNKKPSVLMPSWVLSRNSQKGCSFRKVENHCPKAFCSHXYSQWYMQWVLLGIVSDPRALQCSGVGQGRTLEPPITAVYVSAGLFHVPVLMVGRVSCSHPNMSRAPSERIVITWHLVLCTCHXTQVIFKRKIGNLYFXLELSXDQGSRSNHGAGLLFSQHXLGYRTHISAXDKTELXMSGYGGLWVPHLLCHRTWPLGEARCGEFDCTYPMPHGWVWAVGKPQAPLRGWESTVLGMGKPELVRGSLGLRXGWGHGVLMLLDIWAPLRVEEDLRMDFGLVGWGQPVVQSGGREVGRGRKLWLVPAGKVLGLTGIRLGGDCGWEHREAFYRRLGCSSLGKGLLQVSTHESQXKRQSVVPNHLLSWWKVDAXNHTPHLRVGLRLNTFCREECL

>Frame3

LCSLPDPPDKFSKSSVGARTAXPSRSPQHVGDDDYFRANLCCVKVLSVIAPINNMESMSQVYTKHRTKASQMELTTTHCTKNPLCSPIXTXWCSQQLSHIXLSQXXQKLWVEVYYCQXFVSWQFFCLYIRQEKRPCFHLYKRTKLEGXTKWLMKLMRHLHXRIKSQRLHCAVSLENLKRSQLSKXPEESMNYMQPSIKGRLLQLFISVLXXIXKVWLCGCPPMIWVLSSXRPIVHGGXCRPDCTVFSSQLKLSEPPMQRXQQRTMXDNLSNNQKLHQXRPISWQDVTWSWCXIRAFKKVVVKLRCLHGDIPPPPLYQNFKVAGPXKACSCQPSKLVQGRKTLSKRKLNGSFKDCKSTKESLSKLTYHEVDWAVNKPPKRGRGLSSADKAAVREVSSATDEDVDNSKGSRTIRSHQCXCHPGCYPETVKRVVALGRXRTIALKLSVATDIPSGICSGSCWVSLVILGLCSALVLGRAVHWSHLSLLCMCLLGCSMCPCXWLAGXAALILTCLEHQVKGLLSLGIXCFVHVTELRXSSREKLEIFISDWSSAETKAAGVTMELVYYSVSIDWDTEHTFQHKTKQNYRCQVMEGCGSLICSATGHGHLERQDVGSLTALTPCPMDGCGLXGSPRHHSGDGKAQSXAWESRNWFGGLWACDEAGAMGFSCSWTSGHHXESRKIXEWILGLWAGDSLXSRVVGGRWGEEGNSGLSQQGKSLAXQASGLVVIVAGSTERPFTGGXAAALXAKDFSKSPHMNPNERDSLWFQTIYYHGGKWMHKTIPHISGWAXDXIPFAGRNV

>Mus_musculus_CAAA01080929

IVLPYLDLVVQPAAITHLTESIVAKTLGGGILLPIICLLAIFLSLYKTRKKALLPLVQENQAGGLNQMVNEVNETPALKNKISKAALRRVIRKPQEVTAQQITRRVDELYATLYQGSTSPVVHLGPVVDLKGVALWLSTNDWVLSSXRPIVHGGXCRPDCTVFSSQLKLXXXXXXXTTYLTIRNFINKGLFHGKMLPGVGVESELLRRLWXSSDVCMGTFPPPPYIKTLKLQDHERLAPANLPNLCKVEKTLSKRKLNGSFKDCKSTKESLSKLTYHEVDWAVNKPPKRGRGLSSADKAAVREVSSATDEDVDNSKGS

>Myotis_brandtii_EPQ11842

VSSYAEM

>Frame1

XVSHLCKDDNRGQCR

>Frame2

KXATYAKMTTEDNVG

>Frame3

SEPPMQRXQQRTMXD

>Eptesicus_fuscus_ALEH01011989

ISSFAEM

>Eptesicus_fuscus_ALEH01076397

IATFAEM

>ReptileBorna1_YP_009055058

VASYAQM

>ReptileBorna3

VAKDAQM
